# Altered mitochondrial microenvironment at the spotlight of musculoskeletal aging and Alzheimer’s disease

**DOI:** 10.1038/s41598-022-15578-9

**Published:** 2022-07-04

**Authors:** Panagiotis Giannos, Konstantinos Prokopidis, Stuart M. Raleigh, Eirini Kelaiditi, Mathew Hill

**Affiliations:** 1 Society of Meta-research and Biomedical Innovation, London, UK; 2 Department of Life Sciences, Faculty of Natural Sciences, Imperial College London, South Kensington, London, SW7 2AZ UK; 3 Department of Musculoskeletal Biology, Institute of Life Course and Medical Sciences, University of Liverpool, Liverpool, UK; 4 Cardiovascular and Lifestyle Medicine Research Group, Centre for Sport, Exercise and Life Sciences, Coventry University, Coventry, UK; 5 Faculty of Sport, Allied Health and Performance Science, St Mary’s University Twickenham, Twickenham, UK; 6 Centre for Sport, Exercise and Life Sciences, School of Life Sciences, Coventry University, Coventry, UK

**Keywords:** Alzheimer's disease, Ageing, Musculoskeletal system

## Abstract

Emerging evidence has linked Alzheimer’s disease (AD) onset with musculoskeletal aging via a muscle-brain crosstalk mediated by dysregulation of the mitochondrial microenvironment. This study investigated gene expression profiles from skeletal muscle tissues of older healthy adults to identify potential gene biomarkers whose dysregulated expression and protein interactome were involved in AD. Screening of the literature resulted in 12 relevant microarray datasets (GSE25941, GSE28392, GSE28422, GSE47881, GSE47969, GSE59880) in musculoskeletal aging and (GSE4757, GSE5281, GSE16759, GSE28146, GSE48350, GSE84422) in AD. Retrieved differentially expressed genes (DEGs) were used to construct two unique protein–protein interaction networks and clustering gene modules were identified. Overlapping module DEGs in the musculoskeletal aging and AD networks were ranked based on 11 topological algorithms and the five highest-ranked ones were considered as hub genes. The analysis revealed that the dysregulated expression of the mitochondrial microenvironment genes, NDUFAB1, UQCRC1, UQCRFS1, NDUFS3, and MRPL15, overlapped between both musculoskeletal aging and AD networks. Thus, these genes may have a potential role as markers of AD occurrence in musculoskeletal aging. Human studies are warranted to evaluate the functional role and prognostic value of these genes in aging populations with sarcopenia and AD.

## Introduction

Sarcopenia is primarily a geriatric disease characterized by the progressive decrease of muscle mass, muscle function, and physical performance during aging^1^. Globally, the prevalence of sarcopenia in older adults (≥ 60 years of age) is estimated at 8–13% with increasingly poor health outcomes, including disability, dependency, and reduced quality of life, as a result of the rise in aging population^2^. Interestingly, there is emerging evidence of prominent associations between low handgrip strength and slow gait speed with cognitive dysfunction^3–6^. These alterations may be explained by altered neural signals during aging such as denervated muscle fibers in the neuromuscular junction, impaired motor coordination, dopaminergic neuron downregulation, and subsequent loss of gray matter volume^7,8^. Considering that fluctuations in physical performance and muscle function correspond to changes in brain macrostructure, the muscle-brain crosstalk may underpin a common source of perturbations during aging.

Similar to sarcopenia, neurodegenerative disorders are a major cause of disability and dependency that markedly increases with aging^9^. Recent epidemiological evidence suggests a possible association between sarcopenia and incidence of Alzheimer’s disease (AD), one of the most prevalent causes of late-life cognitive impairment^10^. Indeed, it is now becoming recognized that exercise, which can prevent sarcopenia^11^ and musculoskeletal aging^12^, is also protective against memory decline and AD^13^. Increased oxidative stress and dysregulation of endogenous antioxidant mechanisms, neuroinflammatory responses, mitochondrial dysfunction, and impaired proteostasis, are commonly described factors underpinning both AD and sarcopenia^14^. Presently, pharmacological (i.e. cholinesterase inhibitors, *n*-methyl d-aspartate receptor antagonists) and non-pharmacological (i.e. photobiomodulation, physical activity, nutritional interventions, cognitive remediation) treatments have been utilized to alleviate cognitive and musculoskeletal impairment in individuals with AD, however, these are accompanied by limited efficacy and often considerable side effects^15^. Hence, a greater understanding of the muscle-brain crosstalk at the genetic and epigenetic level may aid in the development of targeted therapies to counteract both musculoskeletal and neurological repercussions during aging.

In this study, we utilized an in silico approach to investigate gene expression profiles from skeletal muscle tissues of older adults and brain tissues of patients with AD. This is the first study aimed at unveiling potential gene markers whose dysregulated expression and protein interactome were involved in both musculoskeletal aging and AD.

## Methods

### Collection of microarray datasets

Searching of the literature was performed from inception until November 2021, by screening the National Center for Biotechnology Information (NCBI) Gene Expression Omnibus (GEO) using the following terms: (aging OR old* OR sarcopenia AND skeletal muscle OR musculoskeletal) and (Alzheimer's disease OR AD). A further search was ensued using the National Library of Medicine (NLM) PubMed following the search terms: (differentially expressed genes OR DEGs). Authors (PG and KP) created the search strategy and conducted the screening of the retrieved datasets.

Datasets were filtered based on organism type (*Homo sapiens*), expression profiling (microarray), sample type (skeletal muscle or brain tissue) and condition (aging and AD). No further exclusion criteria pertained to language, geographic region, and baseline characteristics of patients from which tissue sections were retrieved, were applied. Datasets lacking control expression data were excluded.

### Identification of differentially expressed genes

Musculoskeletal samples from older adults (≥ 60 years of age) were compared to those from healthy young adults (≤ 30 years of age), while brain tissues from patients with AD were compared to those from healthy age-matched individuals. Retrieval of DEGs in musculoskeletal aging was performed using ImaGEO via the random effect model for the integration of differential gene expression^16^. In this case, genes with the strongest average effect across all eligible datasets were selected. DEGs following *P* < 0.05 corrected by the Benjamini–Hochberg False Discovery Rate were retrieved as significant and those with Z score > 1.96 were classified as upregulated, while those with Z score < 1.96 as downregulated (both corresponding to a 5% significance level). Retrieval of DEGs in AD was ensued using GEO2R according to the linear models for microarray analysis. Overlapping DEGs following a *P* < 0.05 were classified as significant, and those with a positive log Fold Change (FC) as upregulated and a negative log FC as downregulated^17^. This collective approach was employed to amplify the inclusion of DEGs and their interactions in musculoskeletal aging while attenuating their by-lack association when compared to those retrieved in AD. The heterogeneity magnitude of DEGs was expressed using Cochran’s Q test and Tau squared.

### Construction of protein–protein interaction networks

DEGs from musculoskeletal aging and AD samples were employed to construct two unique networks of encoded proteins using The Search Tool for the Retrieval of Interacting Genes (STRING)^18^. The protein–protein interactions (PPI) within the two networks were inferred using a medium probabilistic confidence score of > 0.4 and predicted with Cytoscape^19^. The use of a moderate cut-off was ensued to increase the coverage of all possible protein interactions without overestimating their precision. Non-interacting proteins were excluded from the networks.

### Identification of clustering modules and hub genes

Highly clustered DEGs or modules in the two PPI networks were identified using the Molecular Complex Detection (MCODE)^20^. Threshold selection was followed by manual inspection of clusters and a cut off resulting in partition of clusters into distinct groups, was considered. Clusters with MCODE score > 15 were classified as significant modules.

The interactome of module DEGs unique to each PPI network was examined using CytoHubba through the convergence of 11 topological algorithms as proposed by Chin et al.^21^, including: Degree, Closeness, Betweenness, Radiality, Stress, EcCentricity, BottleNeck, Edge Percolated Component (EPC), Maximum Neighborhood Component (MNC), Density of Maximum Neighborhood Component (DMNC) and Maximal Clique Centrality (MCC). The top five module DEGs which overlapped in the musculoskeletal aging and AD networks, were considered as hub genes and presented as potential markers of AD occurrence in musculoskeletal aging.

### Consent for publication

Not applicable.

## Results

### Overview of microarray datasets

The literature search using the GEO and PubMed databases yielded 12 microarray datasets (GSE25941^22^, GSE28392^22^, GSE28422^22^, GSE47881^23,24^, GSE47969^24,25^, GSE59880^25–27^ in musculoskeletal aging and GSE4757^28^, GSE5281^29–32^, GSE16759^33^, GSE28146^34^, GSE48350^35–40^, GSE84422^41^ in AD). The former datasets included skeletal muscle tissues (*vastus lateralis*) from healthy young participants (n = 96) and healthy older adults (n = 110). The latter datasets included brain tissues (medial temporal lobe (entorhinal cortex), parietal lobe, primary visual cortex, medial temporal gyrus, superior frontal gyrus, postcentral gyrus, hippocampus, amygdala and nucleus accumbens) from healthy controls (n = 204) and patients with AD (n = 290) (Table S1).

### Differentially expressed genes in musculoskeletal aging and AD

A sum of 1960 musculoskeletal DEGs were retrieved in older adults when compared to younger counterparts (Table S2). Of these, 1262 upregulated and 698 downregulated DEGs were identified. By contrast, a sum of 3837 DEGs were retrieved in AD patients when compared to healthy counterparts, of which 1855 were upregulated and 1982 were downregulated (Table S3). Between these expression profiles, 406 overlapping DEGs were revealed, 1554 being unique to musculoskeletal aging samples and 3431 to AD ones (Table S4).

### Protein–protein interaction networks and modules in musculoskeletal aging and AD

**Figure 1:**
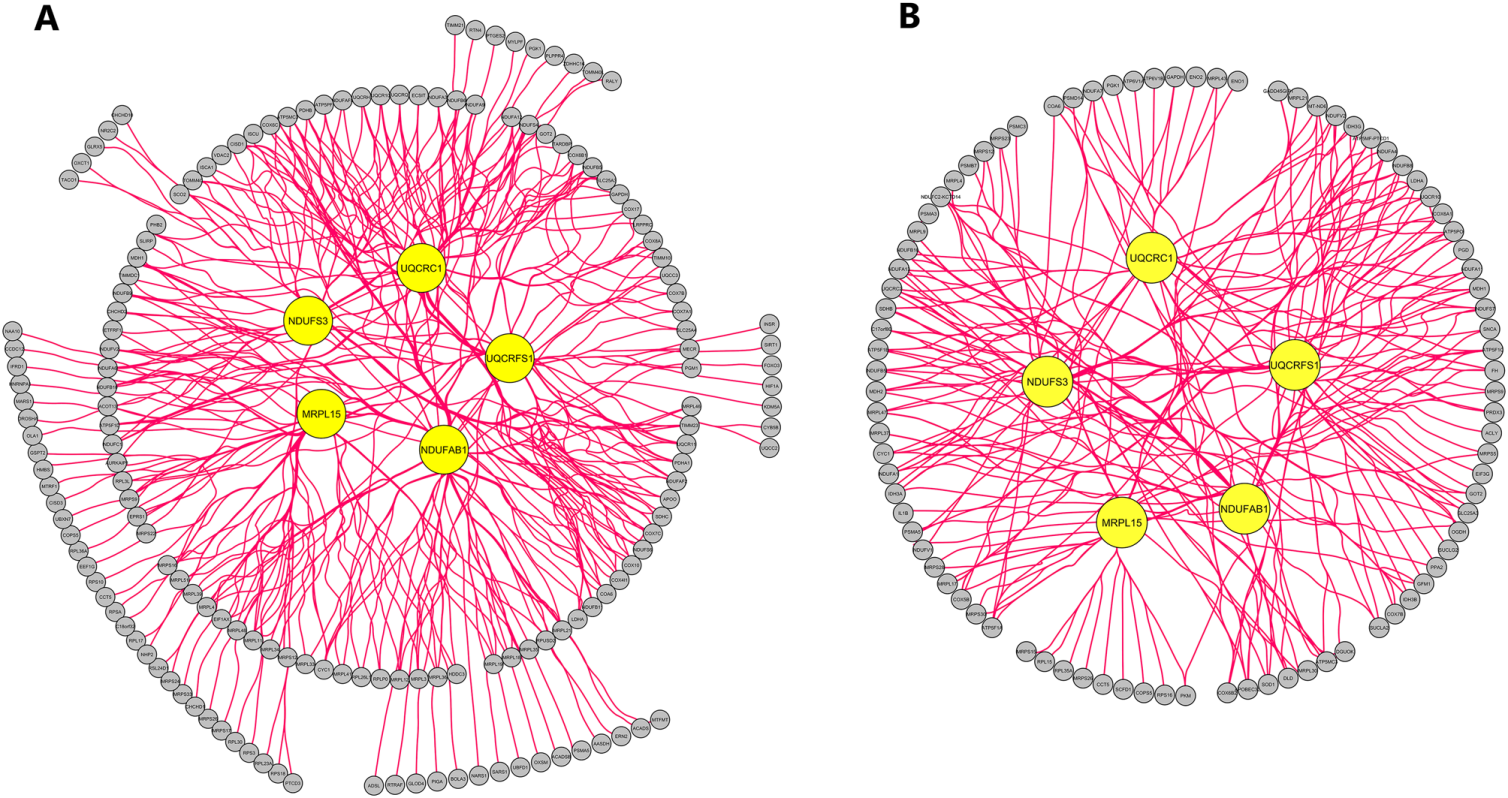
The top five overlapping hub genes of clustering modules in the protein–protein interaction network of differentially expressed genes from (**A**) musculoskeletal aging and (**B**) Alzheimer’s disease. Yellow nodes constitute hub genes. *MRPL15* Mitochondrial ribosomal protein L15, *NDUFAB1* NADH:ubiquinone oxidoreductase subunit AB1, *NDUFS3* NADH:ubiquinone oxidoreductase core subunit S3, *UQCRC1* Ubiquinol-cytochrome c reductase core protein 1, *UQCRFS1* Ubiquinol-cytochrome c reductase, Rieske iron-sulfur polypeptide 1.

Two PPI networks derived from DEGs of musculoskeletal aging and AD were constructed, containing a sum of 1763 and 3492 DEGs along 13,436 and 48,892 interactions, respectively. Two highly clustered gene modules were retrieved in the musculoskeletal aging network and two in the AD one (Tables S5 and S6). The top five hub module DEGs that overlapped between both networks, were identified: NDUFAB1 (NADH:ubiquinone oxidoreductase subunit AB1), UQCRC1 (ubiquinol-cytochrome c reductase core protein 1), UQCRFS1 (ubiquinol-cytochrome c reductase, Rieske iron-sulfur polypeptide 1), NDUFS3 (NADH:ubiquinone oxidoreductase core subunit S3), MRPL15 (mitochondrial ribosomal protein L15) (Table 1, Table S7, Fig. 1).

**Table 1:** The top five ranked and overlapping hub genes according to 11 topological algorithms in the protein–protein interaction networks of musculoskeletal aging and Alzheimer’s disease differentially expressed genes.

Gene ID	Gene name	Musculoskeletal aging		Alzheimer’s disease	
		*P* value	Z-Score	*P* value	logFC
NDUFAB1	NADH:ubiquinone oxidoreductase subunit AB1	3.14E−02	− 3.04	3.59E−16	1.34
UQCRC1	ubiquinol-cytochrome c reductase core protein 1	4.65E−02	− 2.86	1.10E−11	1.64
UQCRFS1	ubiquinol-cytochrome c reductase, Rieske iron-sulfur polypeptide 1	1.31E−06	− 5.88	9.91E−15	1.36
NDUFS3	NADH:ubiquinone oxidoreductase core subunit S3	3.08E−03	− 3.88	8.30E−10	1.09
MRPL15	mitochondrial ribosomal protein L15	9.93E−04	− 4.23	3.71E−07	1.40

*FC* Fold change.

## Discussion

**Figure 2:**
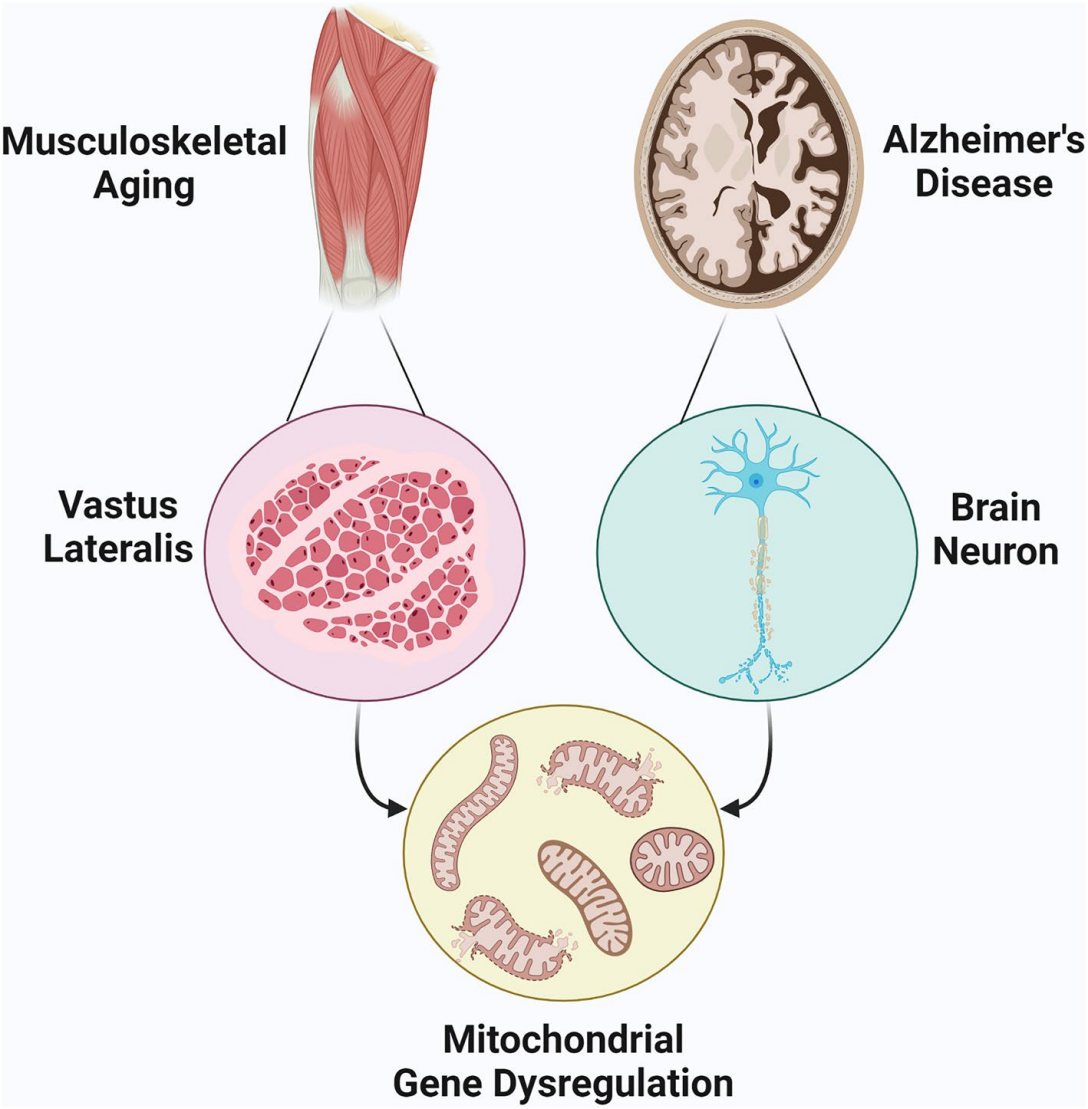
Dysregulated expression of mitochondrial microenvironment genes, NDUFAB1, UQCRC1, UQCRFS1, NDUFS3, and MRPL15, as marker of perturbed muscle-brain crosstalk between musculoskeletal aging and Alzheimer’s disease.

Our analysis on differentially expressed genes of musculoskeletal tissue from older adults and brain tissue samples from patients with AD, revealed two gene clusters in the musculoskeletal aging network and two in the AD network. Multi-algorithmic topological analysis identified five hub genes, NDUFAB1, UQCRC1, UQCRFS1, NDUFS3, and MRPL15, whose dysregulated expression and protein interaction interference overlapped in musculoskeletal aging and AD. A dysregulated opposite tissue expression between the two states was revealed, which hints that musculoskeletal aging genes which might possibly be linked with AD likely acquire aberrations that lead to deranged and opposing expression. These genes may have a potential role as markers of AD occurrence in musculoskeletal aging (Fig. 2).

NDUFAB1 and NDUFS3 are subunits of the NADH dehydrogenase enzyme and constitute central modulators of mitochondrial metabolism in skeletal muscle^42,43^ and the brain^44^. Preliminary research has revealed that ablation of NDUFAB1 in skeletal muscle is linked with dysregulated glucose homeostasis, leading to skeletal muscle insulin resistance^45^. Conversely, increased pyruvate dehydrogenase activity and hence, increased power output via higher adenosine triphosphate (ATP), are all associated with overexpression of NDUFAB1^46^. NDUFAB1 and NDUFS3 have also shown to coordinate mitochondrial respiratory complexes and supercomplexes that enhance ATP synthesis, via the facilitation of electron transfer efficiency and reduction of reactive oxygen species^45,47,48^. Restored NDUFS3 levels in mouse skeletal muscle has equally led to myopathy reversion via mitochondrial complex I regeneration^49^. Interestingly, gene expression analysis from AD profiles has revealed NDUFAB1 and NDUFS3 dysregulation as predictors of AD occurrence and development^50^. Moreover, a positive association between early onset AD with NDUFAB1 and NDUFS3 downregulation has also been suggested with disruption of mitochondrial complex I in brain mitochondria^51,52^. Therefore, the role of NDUFAB1 in skeletal muscle and its connection with brain mitochondrial metabolism may be a key bidirectional association in musculoskeletal aging and AD occurrence^53^.

UQCRC1, UQCRFS1, and MRPL15 are also mitochondrial complex subunits with a prominent role in mitochondrial metabolism^54–56^. Particularly, evidence from in vitro studies has shown that overexpression of UQCRC1 leads to higher phosphorylation of the PI3K/Akt signaling pathway in parallel with cell apoptosis decline via decreased caspase-3 activation^57^. PI3K/Akt is a regulator of muscle hypertrophy, inducing protein synthesis and inhibiting transcriptional mediators of muscle atrophy^58^, whereas caspace-3 has been shown to promote muscle proteolysis via systemic inflammation and activation of the ubiquitin–proteasome system^59^. Muscle disuse and musculoskeletal diseases are both linked with mitochondrial protein expression changes, including UQCRC1 dysregulation^60,61^. Specifically, decreased UQCRC1 content in skeletal muscle is linked with reduced mitochondrial oxidative capacity in a peroxisome proliferator-activated receptor gamma co-activator 1-alpha-dependent manner, leading to muscle fibre atrophy^62^. Moreover, dysfunctions in UQCRC1 and UQCRFS1 are associated with reduced mitochondrial complex III respiratory chain and brain mitochondrial content^56^, leading to disruption of brain mitochondrial bioenergetics^63–65^. Indeed, dysregulation of UQCRC1 and UQCRFS1 are linked with prefrontal cortex degeneration^66^, as shown in blood tissue of patients with AD^67,68^, while recently, a link between MRPL15 with AD diagnosis was revealed^69^. Hence, these alterations imply a potential role of UQCRC1, UQCRFS1, and MRPL15 in molecular modifications underpinning musculoskeletal dysfunction and AD occurrence during aging that warrant further investigation in humans.

### Strengths and limitations

This is the first study to evaluate the possible association of DEGs and their interactome as markers between musculoskeletal aging and AD, using 12 publicly available datasets with a total of 700 tissue samples. In this way, we utilised a multi-algorithmic protein-interaction approach to ensure the subsequential fulfilment of multiple filtering criteria, beyond just gene expression.

Our study is also prone to some limitations. In our analysis, datasets with heterogeneous platforms were not considered, attenuating the broader detection of possible DEGs involved between the two states. However, expression profiling using similar array platforms, as ensued in our study, was employed with a focus on more robust DEGs detection. Moreover, the presence of lab effects has been described to coincide with a known impact in gene profiling through varying array scales which unavoidably underestimates the number of integrated DEGs^70^. Indicative of such phenomenon underlies the contrasting number in DEGs retrieved between the musculoskeletal aging and AD datasets, deferring by 51% in the number total DEGs. Nevertheless, this is a common obstacle observed in the literature and discrepancies in experimental acquisition between labs may predominate, even after normalization^71–74^. This phenomenon was more profound in the AD dataset where the overlap of DEGs was ensued without further batch processing, as to amplify the inclusion of potential DEGs and their interactions in musculoskeletal aging. Moreover, controlling for hidden confounders in modelling gene expression such as demographic traits (sex, age, race), medical comorbidities (i.e., diabetes mellitus, stroke), prescription history (e.g. cholinesterase inhibitors, which are known to modulate mitochondrial function), onset of AD disease and diagnostic modality or evaluation of RNA integrity and post-mortem interval in the included datasets was not possible, based on the lack of availability of such data in the gene expression datasets and their respective individual samples. Likewise, gene expression from different brain regions in the AD group was integrated since there was a scarcity of sufficient data on any a particular brain region. The rationale behind this was to avoid the amplification of detection of differences, which could emphasise statistical differences that may not be clinically relevant (i.e., by chance).

Overall, we identified markers at a transcriptomic level that may modulate the muscle-brain crosstalk and perhaps link age-related musculoskeletal decline and increased occurrence of AD. However, to unveil the underlying molecular mechanisms of these markers and how these could translate to exercise science and sarcopenia in the broader AD field, further bioinformatic confirmation (such as gene co-expression network analysis) and experimental validation are required.

## Conclusions

Age-related musculoskeletal decline and increased occurrence of AD are a global challenge. Studies focusing on the identification of key genetic markers that modulate the muscle-brain crosstalk could provide valuable insight on the relationship between musculoskeletal aging and AD, which could potentiate the development of effective pharmacological therapies or non-pharmacological interventions such as personalised exercise. Our findings revealed that the dysregulated expression of overlapping hub genes, NDUFAB1, UQCRC1, UQCRFS1, NDUFS3, and MRPL15 signified multi-algorithmic topological significance among DEGs from musculoskeletal aging and AD samples, suggesting a prominent link of the mitochondrial microenvironment between these two states. Future experimental human studies are warranted to validate the functional role and the prognostic value of these genes in musculoskeletal aging and AD occurrence.

## Supplementary Information

Supplementary Information 1.

Supplementary Information 2.

## Data Availability

Publicly available datasets were analyzed in this study, and these can be found in the National Center for Biotechnology Information (NCBI) Gene Expression Omnibus (GEO) using the accession codes: GSE25941, GSE28392, GSE28422, GSE47881, GSE47969, GSE59880, GSE4757, GSE5281, GSE16759, GSE28146, GSE48350, GSE84422.

## References

[1] Cruz-Jentoft AJ, Bahat G, Bauer J, Boirie Y, Bruyère O, Cederholm T, Cooper C, Landi F, Rolland Y, Sayer AA (2019). Sarcopenia: Revised European consensus on definition and diagnosis. Age Ageing.

[2] Shafiee G, Keshtkar A, Soltani A, Ahadi Z, Larijani B, Heshmat R (2017). Prevalence of sarcopenia in the world: A systematic review and meta-analysis of general population studies. J. Diabetes Metab. Disord..

[3] Callisaya ML, Blizzard CL, Wood AG, Thrift AG, Wardill T, Srikanth VK (2015). Longitudinal relationships between cognitive decline and gait slowing: The Tasmanian Study of Cognition and Gait. J. Gerontol. Ser. A Biomed. Sci. Med. Sci..

[4] Liu X, Hou L, Xia X, Liu Y, Zuo Z, Zhang Y, Zhao W, Hao Q, Yue J, Dong B (2020). Prevalence of sarcopenia in multi ethnics adults and the association with cognitive impairment: Findings from West-China health and aging trend study. BMC Geriatr..

[5] McGrath R, Vincent BM, Hackney KJ, Robinson-Lane SG, Downer B, Clark BC (2020). The longitudinal associations of handgrip strength and cognitive function in aging Americans. J. Am. Med. Dir. Assoc..

[6] Sternäng O, Reynolds CA, Finkel D, Ernsth-Bravell M, Pedersen NL, Dahl Aslan AK (2016). Grip strength and cognitive abilities: Associations in old age. J. Gerontol. Ser. B Psychol. Sci. Soc. Sci..

[7] Yu JH, Kim RE, Jung J-M, Park SY, Lee DY, Cho HJ, Kim NH, Yoo HJ, Seo JA, Kim SG (2021). Sarcopenia is associated with decreased gray matter volume in the parietal lobe: A longitudinal cohort study. BMC Geriatr..

[8] Kwon YN, Yoon SS (2017). Sarcopenia: Neurological point of view. J. Bone Metab..

[9] Bai A, Xu W, Sun J, Liu J, Deng X, Wu L, Zou X, Zuo J, Zou L, Liu Y (2021). Associations of sarcopenia and its defining components with cognitive function in community-dwelling oldest old. BMC Geriatr..

[10] Beeri MS, Leugrans SE, Delbono O, Bennett DA, Buchman AS (2021). Sarcopenia is associated with incident Alzheimer's dementia, mild cognitive impairment, and cognitive decline. J. Am. Geriatr. Soc..

[11] Ni H-J, Hsu T-F, Chen L-K, Chou H-L, Tung H-H, Chow L-H, Chen Y-C (2022). Effects of exercise programs in older adults with muscle wasting: A systematic review and meta-analysis: Effects of exercise programs in muscle wasting. Arch. Gerontol. Geriatr..

[12] Cartee GD, Hepple RT, Bamman MM, Zierath JR (2016). Exercise promotes healthy aging of skeletal muscle. Cell Metab..

[13] Raleigh SM, Cullen T, Raleigh SM (2021). Alzheimer’s disease, epigenetics, and exercise. Epigenetics of Exercise and Sports.

[14] Van Bulck M, Sierra-Magro A, Alarcon-Gil J, Perez-Castillo A, Morales-Garcia JA (2019). Novel approaches for the treatment of Alzheimer’s and Parkinson’s disease. Int. J. Mol. Sci..

[15] Szeto JYY, Lewis SJG (2016). Current treatment options for Alzheimer’s disease and Parkinson’s disease dementia. Curr. Neuropharmacol..

[16] Toro-Domínguez D, Martorell-Marugán J, López-Domínguez R, García-Moreno A, González-Rumayor V, Alarcón-Riquelme ME, Carmona-Sáez P (2019). ImaGEO: Integrative gene expression meta-analysis from GEO database. Bioinformatics.

[17] Barrett T, Wilhite SE, Ledoux P, Evangelista C, Kim IF, Tomashevsky M, Marshall KA, Phillippy KH, Sherman PM, Holko M (2012). NCBI GEO: Archive for functional genomics data sets—Update. Nucleic Acids Res..

[18] Szklarczyk D, Gable AL, Lyon D, Junge A, Wyder S, Huerta-Cepas J, Simonovic M, Doncheva NT, Morris JH, Bork P (2019). STRING v11: Protein–protein association networks with increased coverage, supporting functional discovery in genome-wide experimental datasets. Nucleic Acids Res..

[19] Shannon P, Markiel A, Ozier O, Baliga NS, Wang JT, Ramage D, Amin N, Schwikowski B, Ideker T (2003). Cytoscape: A software environment for integrated models of biomolecular interaction networks. Genome Res..

[20] Bader GD, Hogue CW (2003). An automated method for finding molecular complexes in large protein interaction networks. BMC Bioinform..

[21] Chin C-H, Chen S-H, Wu H-H, Ho C-W, Ko M-T, Lin C-Y (2014). cytoHubba: Identifying hub objects and sub-networks from complex interactome. BMC Syst. Biol..

[22] Raue U, Trappe TA, Estrem ST, Qian H-R, Helvering LM, Smith RC, Trappe S (2012). Transcriptome signature of resistance exercise adaptations: Mixed muscle and fiber type specific profiles in young and old adults. J. Appl. Physiol..

[23] Phillips BE, Williams JP, Gustafsson T, Bouchard C, Rankinen T, Knudsen S, Smith K, Timmons JA, Atherton PJ (2013). Molecular networks of human muscle adaptation to exercise and age. PLoS Genet..

[24] Timmons JA, Atherton PJ, Larsson O, Sood S, Blokhin IO, Brogan RJ, Volmar C-H, Josse AR, Slentz C, Wahlestedt C (2018). A coding and non-coding transcriptomic perspective on the genomics of human metabolic disease. Nucleic Acids Res..

[25] Sood S, Gallagher IJ, Lunnon K, Rullman E, Keohane A, Crossland H, Phillips BE, Cederholm T, Jensen T, van Loon LJ (2015). A novel multi-tissue RNA diagnostic of healthy ageing relates to cognitive health status. Genome Biol..

[26] Timmons JA, Knudsen S, Rankinen T, Koch LG, Sarzynski M, Jensen T, Keller P, Scheele C, Vollaard NB, Nielsen S (2010). Using molecular classification to predict gains in maximal aerobic capacity following endurance exercise training in humans. J. Appl. Physiol..

[27] Keller P, Vollaard NB, Gustafsson T, Gallagher IJ, Sundberg CJ, Rankinen T, Britton SL, Bouchard C, Koch LG, Timmons JA (2011). A transcriptional map of the impact of endurance exercise training on skeletal muscle phenotype. J. Appl. Physiol..

[28] Dunckley T, Beach TG, Ramsey KE, Grover A, Mastroeni D, Walker DG, LaFleur BJ, Coon KD, Brown KM, Caselli R (2006). Gene expression correlates of neurofibrillary tangles in Alzheimer's disease. Neurobiol. Aging.

[29] Liang WS, Dunckley T, Beach TG, Grover A, Mastroeni D, Walker DG, Caselli RJ, Kukull WA, McKeel D, Morris JC (2007). Gene expression profiles in anatomically and functionally distinct regions of the normal aged human brain. Physiol. Genom..

[30] Liang WS, Reiman EM, Valla J, Dunckley T, Beach TG, Grover A, Niedzielko TL, Schneider LE, Mastroeni D, Caselli R (2008). Alzheimer's disease is associated with reduced expression of energy metabolism genes in posterior cingulate neurons. Proc. Natl. Acad. Sci..

[31] Readhead B, Haure-Mirande J-V, Funk CC, Richards MA, Shannon P, Haroutunian V, Sano M, Liang WS, Beckmann ND, Price ND (2018). Multiscale analysis of independent Alzheimer’s cohorts finds disruption of molecular, genetic, and clinical networks by human herpesvirus. Neuron.

[32] Liang WS, Dunckley T, Beach TG, Grover A, Mastroeni D, Ramsey K, Caselli RJ, Kukull WA, McKeel D, Morris JC (2008). Altered neuronal gene expression in brain regions differentially affected by Alzheimer's disease: A reference data set. Physiol. Genom..

[33] Nunez-Iglesias J, Liu C-C, Morgan TE, Finch CE, Zhou XJ (2010). Joint genome-wide profiling of miRNA and mRNA expression in Alzheimer's disease cortex reveals altered miRNA regulation. PLoS ONE.

[34] Blalock EM, Buechel HM, Popovic J, Geddes JW, Landfield PW (2011). Microarray analyses of laser-captured hippocampus reveal distinct gray and white matter signatures associated with incipient Alzheimer's disease. J. Chem. Neuroanat..

[35] Berchtold NC, Cribbs DH, Coleman PD, Rogers J, Head E, Kim R, Beach T, Miller C, Troncoso J, Trojanowski JQ (2008). Gene expression changes in the course of normal brain aging are sexually dimorphic. Proc. Natl. Acad. Sci..

[36] Berchtold NC, Coleman PD, Cribbs DH, Rogers J, Gillen DL, Cotman CW (2013). Synaptic genes are extensively downregulated across multiple brain regions in normal human aging and Alzheimer's disease. Neurobiol. Aging.

[37] Cribbs DH, Berchtold NC, Perreau V, Coleman PD, Rogers J, Tenner AJ, Cotman CW (2012). Extensive innate immune gene activation accompanies brain aging, increasing vulnerability to cognitive decline and neurodegeneration: A microarray study. J. Neuroinflamm..

[38] Astarita G, Jung K-M, Berchtold NC, Nguyen VQ, Gillen DL, Head E, Cotman CW, Piomelli D (2010). Deficient liver biosynthesis of docosahexaenoic acid correlates with cognitive impairment in Alzheimer's disease. PLoS ONE.

[39] Blair LJ, Nordhues BA, Hill SE, Scaglione KM, O’Leary JC, Fontaine SN, Breydo L, Zhang B, Li P, Wang L (2013). Accelerated neurodegeneration through chaperone-mediated oligomerization of tau. J. Clin. Investig..

[40] Sárvári M, Hrabovszky E, Kalló I, Solymosi N, Likó I, Berchtold N, Cotman C, Liposits Z (2012). Menopause leads to elevated expression of macrophage-associated genes in the aging frontal cortex: Rat and human studies identify strikingly similar changes. J. Neuroinflamm..

[41] Wang M, Roussos P, McKenzie A, Zhou X, Kajiwara Y, Brennand KJ, De Luca GC, Crary JF, Casaccia P, Buxbaum JD (2016). Integrative network analysis of nineteen brain regions identifies molecular signatures and networks underlying selective regional vulnerability to Alzheimer’s disease. Genome Med..

[42] Chae S, Kim S-J, Do Koo Y, Lee JH, Kim H, Ahn BY, Ha Y-C, Kim Y-H, Jang MG, Koo K-H (2018). A mitochondrial proteome profile indicative of type 2 diabetes mellitus in skeletal muscles. Exp. Mol. Med..

[43] Guerrero-Castillo S, Baertling F, Kownatzki D, Wessels HJ, Arnold S, Brandt U, Nijtmans L (2017). The assembly pathway of mitochondrial respiratory chain complex I. Cell Metab..

[44] Guo X, Park JE, Gallart-Palau X, Sze SK (2020). Oxidative damage to the TCA cycle enzyme MDH1 dysregulates bioenergetic enzymatic activity in the aged murine brain. J. Proteome Res..

[45] Zhang R, Hou T, Cheng H, Wang X (2019). NDUFAB1 protects against obesity and insulin resistance by enhancing mitochondrial metabolism. FASEB J..

[46] Kasper JD, Meyer RA, Beard DA, Wiseman RW (2019). Effects of altered pyruvate dehydrogenase activity on contracting skeletal muscle bioenergetics. Am. J. Physiol. Regul. Integr. Comp. Physiol..

[47] Luo N, Yue F, Jia Z, Chen J, Deng Q, Zhao Y, Kuang S (2021). Reduced electron transport chain complex I protein abundance and function in Mfn2-deficient myogenic progenitors lead to oxidative stress and mitochondria swelling. FASEB J..

[48] Hou T, Zhang R, Jian C, Ding W, Wang Y, Ling S, Ma Q, Hu X, Cheng H, Wang X (2019). NDUFAB1 confers cardio-protection by enhancing mitochondrial bioenergetics through coordination of respiratory complex and supercomplex assembly. Cell Res..

[49] Pereira CV, Peralta S, Arguello T, Bacman SR, Diaz F, Moraes CT (2020). Myopathy reversion in mice after restauration of mitochondrial complex I. EMBO Mol. Med..

[50] Wang Z, Yan X, Zhao C (2017). Dynamical differential networks and modules inferring disrupted genes associated with the progression of Alzheimer's disease. Exp. Ther. Med..

[51] Zhang X, Gao X, Coots RA, Conn CS, Liu B, Qian S-B (2015). Translational control of the cytosolic stress response by mitochondrial ribosomal protein L18. Nat. Struct. Mol. Biol..

[52] Adav SS, Park JE, Sze SK (2019). Quantitative profiling brain proteomes revealed mitochondrial dysfunction in Alzheimer’s disease. Mol. Brain.

[53] Lopez Sanchez MIG, Krüger A, Shiriaev DI, Liu Y, Rorbach J (2021). Human mitoribosome biogenesis and its emerging links to disease. Int. J. Mol. Sci..

[54] Haque ME, Grasso D, Miller C, Spremulli LL, Saada A (2008). The effect of mutated mitochondrial ribosomal proteins S16 and S22 on the assembly of the small and large ribosomal subunits in human mitochondria. Mitochondrion.

[55] Sato T, Chang H-C, Bayeva M, Shapiro JS, Ramos-Alonso L, Kouzu H, Jiang X, Liu T, Yar S, Sawicki KT (2018). mRNA-binding protein tristetraprolin is essential for cardiac response to iron deficiency by regulating mitochondrial function. Proc. Natl. Acad. Sci..

[56] Burska D, Stiburek L, Krizova J, Vanisova M, Martinek V, Sladkova J, Zamecnik J, Honzik T, Zeman J, Hansikova H (2021). Homozygous missense mutation in UQCRC2 associated with severe encephalomyopathy, mitochondrial complex III assembly defect and activation of mitochondrial protein quality control. Biochim. Biophys. Acta (BBA) Mol. Basis Dis..

[57] Yi T, Wu X, Li H (2020). Ubiquinol-cytochrome c reductase core protein 1 overexpression protects H9c2 cardiac cells against mimic ischemia/reperfusion injury through PI3K/Akt/GSK-3β pathway. Biochem. Biophys. Res. Commun..

[58] Schiaffino S, Mammucari C (2011). Regulation of skeletal muscle growth by the IGF1-Akt/PKB pathway: Insights from genetic models. Skelet. Muscle.

[59] Zhu S, Nagashima M, Khan MA, Yasuhara S, Kaneki M, Martyn JJ (2013). Lack of caspase-3 attenuates immobilization-induced muscle atrophy and loss of tension generation along with mitigation of apoptosis and inflammation. Muscle Nerve.

[60] Flück M, Li R, Valdivieso P, Linnehan RM, Castells J, Tesch P, Gustafsson T (2014). Early changes in costameric and mitochondrial protein expression with unloading are muscle specific. BioMed Res. Int..

[61] Unni S, Thiyagarajan S, Bharath MS, Padmanabhan B (2019). Tryptophan oxidation in the UQCRC1 subunit of mitochondrial complex III (ubiquinol-cytochrome C reductase) in a mouse model of myodegeneration causes large structural changes in the complex: A molecular dynamics simulation study. Sci. Rep..

[62] Kristensen JM, Skov V, Petersson SJ, Ørtenblad N, Wojtaszewski JF, Beck-Nielsen H, Højlund K (2014). A PGC-1α-and muscle fibre type-related decrease in markers of mitochondrial oxidative metabolism in skeletal muscle of humans with inherited insulin resistance. Diabetologia.

[63] Hu WH, Hausmann ON, Yan MS, Walters WM, Wong PK, Bethea JR (2002). Identification and characterization of a novel Nogo-interacting mitochondrial protein (NIMP). J. Neurochem..

[64] Kriaucionis S, Paterson A, Curtis J, Guy J, MacLeod N, Bird A (2006). Gene expression analysis exposes mitochondrial abnormalities in a mouse model of Rett syndrome. Mol. Cell. Biol..

[65] Shan W, Li J, Xu W, Li H, Zuo Z (2019). Critical role of UQCRC1 in embryo survival, brain ischemic tolerance and normal cognition in mice. Cell. Mol. Life Sci..

[66] Palmfeldt J, Henningsen K, Eriksen SA, Müller HK, Wiborg O (2016). Protein biomarkers of susceptibility and resilience to stress in a rat model of depression. Mol. Cell. Neurosci..

[67] Rahman MR, Islam T, Zaman T, Shahjaman M, Karim MR, Huq F, Quinn JM, Holsinger RD, Gov E, Moni MA (2020). Identification of molecular signatures and pathways to identify novel therapeutic targets in Alzheimer's disease: Insights from a systems biomedicine perspective. Genomics.

[68] Salat DH, Kaye JA, Janowsky JS (2001). Selective preservation and degeneration within the prefrontal cortex in aging and Alzheimer disease. Arch. Neurol..

[69] Gao L, Li J, Yan M, Aili M (2021). Methylation factor MRPL15 identified as a potential biological target in Alzheimer’s disease. Aging (Albany NY).

[70] Lyu Y, Li Q (2016). A semi-parametric statistical model for integrating gene expression profiles across different platforms. BMC bioinform..

[71] Johnson WE, Li C, Rabinovic A (2007). Adjusting batch effects in microarray expression data using empirical Bayes methods. Biostatistics.

[72] Dillies M-A, Rau A, Aubert J, Hennequet-Antier C, Jeanmougin M, Servant N, Keime C, Marot G, Castel D, Estelle J (2013). A comprehensive evaluation of normalization methods for Illumina high-throughput RNA sequencing data analysis. Brief. Bioinform..

[73] Hansen KD, Irizarry RA, Wu Z (2012). Removing technical variability in RNA-seq data using conditional quantile normalization. Biostatistics.

[74] Roberts A, Trapnell C, Donaghey J, Rinn JL, Pachter L (2011). Improving RNA-Seq expression estimates by correcting for fragment bias. Genome Biol..

