# Supplementary material for: Altered mitochondrial microenvironment at the spotlight of musculoskeletal aging and Alzheimer’s disease
Source: Sci Rep. 2022 Jul 4;12:11290. doi: 10.1038/s41598-022-15578-9 (PMC9253146; doi:10.1038/s41598-022-15578-9)
Supplement: Supplementary file 1 — Supplementary Information 1. [file 41598_2022_15578_MOESM1_ESM.docx]

**Supplementary Material**

**Table S1.** Characteristics of the gene expression datasets included in the analysis.

| **Gene expression datasets** | **Controls** | | | **Experiments** | | | **Tissue sample** | **Microarray**  **platform** |
| --- | --- | --- | --- | --- | --- | --- | --- | --- |
|  | ***n^*^*** | **age** | **sex** | ***n^*^*** | **age** | **sex** |  |  |
| *Musculoskeletal aging* | | | | | | | | |
| GSE25941 | 15 | 24  -  26 | M/F | 21 | 77  -  79 | M/F | Vastus lateralis | GPL570 |
| GSE28392 | 20 | 21  -  25 | F | 14 | 84  -  86 | F | Vastus lateralis | GPL570 |
| GSE28422 | 30 | 23  -  25 | M/F | 24 | 83  -  85 | M/F | Vastus lateralis | GPL570 |
| GSE47881 | 10 | 18  -  30 | M/F | 16 | 60  -  75 | M/F | Vastus lateralis | GPL570 |
| GSE47969 | 6 | 24  -  30 | M/F | 22 | 60  -  68 | M/F | Vastus lateralis | GPL570 |
| GSE59880 | 15 | 19  -  28 | M | 13 | 59  -  77 | M | Vastus lateralis | GPL570 |
|  | | | | | | | | |
| *Alzheimer’s disease* | | | | | | | | |
| GSE4757 | 10 | 72  -  88 |  | 10 | 77  -  93 |  | Entorhinal cortex | GPL570 |
| GSE5281 | 74 | 63  -  102 | M/F | 87 | 68  -  >90 | M/F | Entorhinal cortex, Hippocampus, Medial temporal gyrus, Posterior cingulate, Superior frontal gyrus, Primary visual cortex | GPL570 |
| GSE16759 | 4 | 90  -  95 | M/F | 4 | 82  -  92 | M/F | Parietal lobe | GPL570 |
| GSE28146 | 8 | 75  -  97 | M/F | 22 | 83  -  101 | M/F | Hippocampus | GPL570 |
| GSE48350 | 93 | 60  -  99 | M/F | 80 | 60  -  95 | M/F | Entorhinal cortex, Hippocampus, Postcentral gyrus,  Superior frontal gyrus | GPL570 |
| GSE84422 | 15 | 64  -  102 | M/F | 87 | 62  -  102 | F | Amygdala, Nucleus accumbens | GPL570 |

***^*^*** *Sample size.*

**Table S4.** Overlapping differentially expressed genes of brain samples between musculoskeletal aging and Alzheimer’s disease.

.

| **Gene ID** | **Musculoskeletal aging** | | **Alzheimer’s disease** | | **Gene name** |
| --- | --- | --- | --- | --- | --- |
|  | **P-value** | **Z-score** | **P-value** | **Log FC** |  |
| AAGAB | 8.88E-03 | 3.53 | 2.4E-07 | 1.14 | alpha and gamma adaptin binding protein |
| ABCB4 | 1.66E-03 | 4.07 | 1.4E-02 | 1.42 | ATP binding cassette subfamily B member 4 |
| ABHD14A-ACY1 | 1.56E-05 | -5.32 | 2.0E-03 | -1.63 | ABHD14A-ACY1 readthrough |
| ACADSB | 4.78E-02 | 2.85 | 7.4E-04 | 1.90 | acyl-CoA dehydrogenase short/branched chain |
| ACTB | 2.43E-02 | 3.14 | 3.4E-10 | 1.51 | actin beta |
| ADAMTSL4 | 2.75E-08 | 6.60 | 2.8E-07 | -1.10 | ADAMTS like 4 |
| ADIRF | 8.78E-04 | 4.26 | 5.4E-03 | -1.14 | adipogenesis regulatory factor |
| AGAP9 | 4.38E-02 | 2.89 | 5.4E-12 | -1.61 | ArfGAP with GTPase domain, ankyrin repeat and PH domain 9 |
| AGBL3 | 6.74E-03 | 3.62 | 9.7E-03 | -1.12 | AGBL carboxypeptidase 3 |
| AHNAK2 | 1.01E-02 | 3.48 | 1.2E-08 | 1.18 | AHNAK nucleoprotein 2 |
| ALPK2 | 7.83E-08 | 6.41 | 1.3E-02 | -1.60 | alpha kinase 2 |
| ANAPC16 | 3.55E-02 | 2.98 | 1.0E-12 | -1.09 | anaphase promoting complex subunit 16 |
| ANK3 | 2.18E-04 | 4.65 | 5.0E-09 | 1.16 | ankyrin 3 |
| ANKRD12 | 1.12E-06 | 5.91 | 3.4E-10 | -1.54 | ankyrin repeat domain 12 |
| ANKRD13A | 2.46E-02 | 3.14 | 9.0E-15 | -1.48 | ankyrin repeat domain 13A |
| ANKRD36 | 1.11E-02 | 3.44 | 7.1E-15 | -1.73 | ankyrin repeat domain 36 |
| ANKRD36B | 2.40E-05 | 5.22 | 2.1E-06 | -1.07 | ankyrin repeat domain 36B |
| ANKRD6 | 9.36E-03 | 3.51 | 2.3E-02 | -1.10 | ankyrin repeat domain 6 |
| ANP32B | 4.19E-02 | 2.91 | 7.2E-18 | -1.53 | acidic nuclear phosphoprotein 32 family member B |
| ANXA2 | 1.90E-02 | -3.24 | 4.7E-02 | -1.19 | annexin A2 |
| ANXA6 | 1.13E-02 | -3.44 | 7.5E-15 | 1.51 | annexin A6 |
| API5 | 1.68E-02 | 3.29 | 1.2E-07 | 1.41 | apoptosis inhibitor 5 |
| APLP2 | 3.81E-02 | 2.95 | 1.0E-09 | 1.06 | amyloid beta precursor like protein 2 |
| APOO | 1.70E-07 | -6.28 | 1.9E-13 | 1.47 | apolipoprotein O |
| ARHGAP20 | 3.25E-02 | 3.02 | 2.6E-02 | 1.09 | Rho GTPase activating protein 20 |
| ARHGAP32 | 2.45E-02 | 3.14 | 5.7E-08 | 1.02 | Rho GTPase activating protein 32 |
| ARHGEF9 | 2.09E-02 | -3.20 | 2.7E-13 | 1.24 | Cdc42 guanine nucleotide exchange factor 9 |
| ARMCX4 | 4.04E-07 | 6.12 | 1.0E-05 | 1.12 | armadillo repeat containing X-linked 4 |
| ARSG | 4.56E-02 | 2.87 | 1.2E-04 | -0.19 | arylsulfatase G |
| ATG2B | 3.15E-03 | 3.87 | 4.0E-08 | 1.23 | autophagy related 2B |
| ATL1 | 4.28E-03 | 3.77 | 1.3E-09 | 1.16 | atlastin GTPase 1 |
| ATP5MC3 | 1.62E-06 | -5.84 | 1.8E-06 | 1.23 | ATP synthase membrane subunit c locus 3 |
| ATP5MJ | 1.42E-03 | -4.12 | 5.7E-14 | 1.11 | ATP synthase membrane subunit j |
| ATP6AP1L | 1.37E-03 | 4.14 | 4.0E-02 | -1.64 | ATPase H+ transporting accessory protein 1 like (pseudogene) |
| ATP6V0B | 1.93E-02 | -3.23 | 1.3E-06 | 1.12 | ATPase H+ transporting V0 subunit b |
| ATP6V0E1 | 6.15E-03 | 3.65 | 4.1E-10 | -1.32 | ATPase H+ transporting V0 subunit e1 |
| ATP8B1 | 5.61E-06 | 5.54 | 2.7E-17 | -1.40 | ATPase phospholipid transporting 8B1 |
| ATP8B4 | 1.08E-04 | 4.83 | 2.2E-02 | -1.40 | ATPase phospholipid transporting 8B4 (putative) |
| ATRX | 1.11E-04 | 4.82 | 1.7E-09 | -0.06 | ATRX chromatin remodeler |
| AZI2 | 2.86E-02 | 3.07 | 3.9E-09 | 1.12 | 5-azacytidine induced 2 |
| BAZ1A | 2.10E-03 | 4.00 | 6.2E-10 | -1.37 | bromodomain adjacent to zinc finger domain 1A |
| BCL6 | 1.45E-03 | 4.12 | 4.5E-08 | -1.13 | BCL6 transcription repressor |
| BHLHE40 | 3.04E-02 | 3.05 | 3.9E-04 | -1.28 | basic helix-loop-helix family member e40 |
| BRWD1 | 7.93E-03 | 3.56 | 3.8E-14 | 1.53 | bromodomain and WD repeat domain containing 1 |
| C12orf29 | 1.86E-02 | 3.25 | 6.2E-04 | 1.16 | chromosome 12 open reading frame 29 |
| C1QB | 6.25E-05 | 4.98 | 6.4E-03 | -1.13 | complement C1q B chain |
| C1S | 5.04E-06 | 5.57 | 4.1E-03 | -1.42 | complement C1s |
| C3 | 2.77E-04 | 4.59 | 1.8E-02 | -1.10 | complement C3 |
| C6orf89 | 1.44E-03 | 4.12 | 2.2E-05 | -1.05 | chromosome 6 open reading frame 89 |
| CACFD1 | 5.93E-03 | -3.67 | 2.9E-02 | -1.15 | calcium channel flower domain containing 1 |
| CAMK2B | 2.76E-02 | 3.09 | 7.1E-07 | 1.12 | calcium/calmodulin dependent protein kinase II beta |
| CBR1 | 7.17E-04 | -4.32 | 6.8E-03 | -1.29 | carbonyl reductase 1 |
| CCDC69 | 1.28E-02 | -3.39 | 4.2E-03 | 1.58 | coiled-coil domain containing 69 |
| CCN2 | 1.87E-08 | 6.67 | 2.1E-02 | -1.48 | cellular communication network factor 2 |
| CCNC | 3.53E-02 | 2.99 | 1.6E-09 | -1.26 | cyclin C |
| CCT5 | 3.41E-02 | 3.00 | 2.1E-10 | 1.60 | chaperonin containing TCP1 subunit 5 |
| CCT7 | 4.95E-02 | -2.84 | 4.3E-10 | 1.34 | chaperonin containing TCP1 subunit 7 |
| CDC40 | 2.91E-02 | 3.07 | 3.9E-13 | 1.16 | cell division cycle 40 |
| CEP290 | 1.49E-03 | 4.11 | 4.2E-02 | -1.14 | centrosomal protein 290 |
| CHCHD2 | 3.79E-02 | -2.95 | 3.2E-12 | 1.11 | coiled-coil-helix-coiled-coil-helix domain containing 2 |
| CIART | 1.89E-03 | 4.03 | 2.8E-02 | -1.23 | circadian associated repressor of transcription |
| CIRBP | 4.29E-02 | 2.90 | 3.9E-10 | 1.24 | cold inducible RNA binding protein |
| CLIP1 | 4.48E-02 | 2.88 | 1.8E-05 | -1.17 | CAP-Gly domain containing linker protein 1 |
| CLN8 | 1.83E-02 | -3.25 | 2.6E-02 | 1.01 | CLN8 transmembrane ER and ERGIC protein |
| CMSS1 | 1.07E-03 | 4.21 | 6.8E-16 | 1.09 | cms1 ribosomal small subunit homolog |
| COA6 | 2.10E-03 | -4.00 | 3.1E-02 | 1.14 | cytochrome c oxidase assembly factor 6 |
| COL1A2 | 3.58E-02 | 2.98 | 1.6E-07 | -1.60 | collagen type I alpha 2 chain |
| COPS5 | 8.76E-07 | -5.96 | 1.0E-13 | 1.27 | COP9 signalosome subunit 5 |
| COX7B | 2.62E-02 | 3.12 | 1.3E-11 | 1.16 | cytochrome c oxidase subunit 7B |
| CP | 1.84E-06 | 5.81 | 3.1E-02 | -1.11 | ceruloplasmin |
| CPEB4 | 1.37E-03 | 4.14 | 1.7E-10 | -1.14 | cytoplasmic polyadenylation element binding protein 4 |
| CPM | 7.54E-03 | 3.59 | 4.4E-05 | -1.31 | carboxypeptidase M |
| CRIPT | 6.10E-05 | 4.99 | 7.7E-03 | 1.15 | CXXC repeat containing interactor of PDZ3 domain |
| CUX1 | 2.51E-02 | 3.13 | 1.9E-07 | -1.21 | cut like homeobox 1 |
| CXorf38 | 4.07E-02 | 2.92 | 1.4E-03 | -1.92 | chromosome X open reading frame 38 |
| CYC1 | 1.27E-05 | -5.36 | 1.6E-07 | 1.15 | cytochrome c1 |
| DCLK1 | 1.09E-04 | 4.83 | 1.2E-09 | 1.26 | doublecortin like kinase 1 |
| DDIT4L | 1.52E-04 | -4.74 | 2.5E-07 | -1.05 | DNA damage inducible transcript 4 like |
| DDX1 | 3.74E-02 | -2.96 | 5.0E-09 | 1.12 | DEAD-box helicase 1 |
| DGKG | 2.66E-02 | 3.11 | 2.1E-12 | -1.59 | diacylglycerol kinase gamma |
| DHRS9 | 1.81E-02 | 3.26 | 1.8E-02 | -1.21 | dehydrogenase/reductase 9 |
| DHX36 | 3.56E-03 | 3.83 | 5.4E-09 | 1.13 | DEAH-box helicase 36 |
| DLEU2 | 9.26E-05 | 4.88 | 6.2E-03 | -1.17 | deleted in lymphocytic leukemia 2 |
| DNAJB1 | 1.85E-02 | 3.25 | 8.9E-03 | 1.21 | DnaJ heat shock protein family (Hsp40) member B1 |
| DNPH1 | 1.64E-05 | -5.30 | 5.3E-06 | 1.29 | 2'-deoxynucleoside 5'-phosphate N-hydrolase 1 |
| DPP6 | 3.25E-02 | 3.02 | 7.4E-15 | 1.22 | dipeptidyl peptidase like 6 |
| DPY30 | 3.21E-02 | -3.03 | 1.2E-10 | 1.05 | dpy-30 histone methyltransferase complex regulatory subunit |
| DRD2 | 9.84E-03 | -3.49 | 3.0E-03 | 1.37 | dopamine receptor D2 |
| EBLN2 | 3.19E-04 | 4.54 | 1.2E-03 | -1.90 | endogenous Bornavirus like nucleoprotein 2 |
| EDN1 | 5.34E-06 | 5.55 | 3.2E-02 | -1.18 | endothelin 1 |
| EFCAB7 | 2.28E-03 | 3.97 | 1.6E-05 | 1.03 | EF-hand calcium binding domain 7 |
| EGR1 | 1.89E-02 | 3.24 | 3.0E-17 | 1.58 | early growth response 1 |
| EIF2B3 | 3.74E-02 | -2.96 | 1.1E-16 | 1.38 | eukaryotic translation initiation factor 2B subunit gamma |
| EIF3C | 1.31E-03 | 4.15 | 1.4E-07 | -1.20 | eukaryotic translation initiation factor 3 subunit C |
| ENC1 | 1.93E-02 | 3.23 | 8.3E-09 | 1.44 | ectodermal-neural cortex 1 |
| ENDOG | 7.87E-04 | -4.30 | 6.9E-07 | 1.09 | endonuclease G |
| EPB41L3 | 7.48E-07 | 6.00 | 1.0E-12 | 1.75 | erythrocyte membrane protein band 4.1 like 3 |
| EPDR1 | 2.49E-02 | -3.13 | 1.1E-10 | 1.42 | ependymin related 1 |
| ERV3-2 | 1.01E-03 | 4.22 | 2.5E-02 | -1.14 | endogenous retrovirus group 3 member 2 |
| ESPN | 1.90E-02 | -3.24 | 3.4E-02 | -1.33 | espin |
| EXOC8 | 2.91E-02 | 3.07 | 1.7E-10 | 1.04 | exocyst complex component 8 |
| EXOSC7 | 2.12E-02 | 3.20 | 5.9E-03 | -1.56 | exosome component 7 |
| F5 | 2.42E-02 | 3.15 | 4.5E-03 | -1.29 | coagulation factor V |
| FAM107B | 1.09E-04 | 4.83 | 5.9E-14 | -1.63 | family with sequence similarity 107 member B |
| FAM161A | 2.93E-04 | 4.57 | 1.3E-07 | -1.02 | FAM161 centrosomal protein A |
| FAM171A2 | 5.85E-03 | -3.67 | 3.2E-02 | -1.36 | family with sequence similarity 171 member A2 |
| FAT1 | 4.21E-02 | 2.91 | 7.0E-11 | -1.13 | FAT atypical cadherin 1 |
| FBXO16 | 3.63E-04 | 4.50 | 2.0E-05 | -1.11 | F-box protein 16 |
| FGF13 | 2.12E-02 | 3.20 | 1.2E-08 | 1.33 | fibroblast growth factor 13 |
| FHDC1 | 3.25E-03 | -3.86 | 6.0E-03 | -1.54 | FH2 domain containing 1 |
| FHL2 | 2.82E-02 | -3.08 | 1.8E-14 | 1.31 | four and a half LIM domains 2 |
| FLII | 1.43E-02 | 3.35 | 1.9E-02 | -1.35 | FLII actin remodeling protein |
| FLT1 | 4.21E-02 | 2.91 | 2.0E-06 | -1.32 | fms related receptor tyrosine kinase 1 |
| FMNL2 | 1.44E-02 | 3.35 | 5.4E-12 | -1.17 | formin like 2 |
| FNIP1 | 3.37E-02 | 3.01 | 6.1E-08 | -1.17 | folliculin interacting protein 1 |
| FOXO1 | 4.21E-02 | -2.91 | 3.6E-13 | -1.09 | forkhead box O1 |
| FXYD2 | 2.44E-02 | -3.14 | 4.2E-02 | -1.28 | FXYD domain containing ion transport regulator 2 |
| FXYD6 | 1.95E-02 | 3.23 | 6.1E-06 | 1.06 | FXYD domain containing ion transport regulator 6 |
| GALNT17 | 2.02E-03 | -4.01 | 1.6E-09 | 1.04 | polypeptide N-acetylgalactosaminyltransferase 17 |
| GAPDH | 4.77E-02 | 2.85 | 6.5E-10 | 1.22 | glyceraldehyde-3-phosphate dehydrogenase |
| GBP2 | 8.34E-05 | 4.90 | 8.0E-08 | -1.32 | guanylate binding protein 2 |
| GCH1 | 1.54E-03 | 4.10 | 7.0E-03 | -1.49 | GTP cyclohydrolase 1 |
| GDA | 3.94E-02 | -2.94 | 1.9E-07 | 1.37 | guanine deaminase |
| GET4 | 2.44E-02 | 3.14 | 3.0E-02 | -1.31 | guided entry of tail-anchored proteins factor 4 |
| GINS4 | 1.05E-03 | 4.21 | 4.4E-02 | -1.35 | GINS complex subunit 4 |
| GLOD4 | 8.80E-06 | -5.45 | 3.8E-12 | 1.05 | glyoxalase domain containing 4 |
| GLRX | 1.49E-03 | 4.11 | 2.8E-09 | 1.31 | glutaredoxin |
| GNG2 | 1.39E-02 | 3.36 | 5.4E-07 | 1.19 | G protein subunit gamma 2 |
| GON4L | 5.33E-03 | -3.70 | 3.2E-02 | -1.02 | gon-4 like |
| GOT1 | 2.02E-05 | -5.26 | 1.1E-16 | 1.87 | glutamic-oxaloacetic transaminase 1 |
| GOT2 | 8.61E-06 | 5.45 | 1.9E-14 | 1.35 | glutamic-oxaloacetic transaminase 2 |
| GPAM | 2.06E-02 | 3.21 | 1.1E-08 | -1.04 | glycerol-3-phosphate acyltransferase, mitochondrial |
| GPATCH2L | 3.26E-05 | 5.14 | 3.0E-05 | -1.10 | G-patch domain containing 2 like |
| GRAMD1C | 1.38E-02 | -3.37 | 9.3E-11 | -1.21 | GRAM domain containing 1C |
| H2AC14 | 1.93E-02 | 3.23 | 1.6E-02 | -1.23 | H2A clustered histone 14 |
| HBP1 | 3.49E-02 | -2.99 | 1.3E-14 | -1.86 | HMG-box transcription factor 1 |
| HIGD1B | 1.34E-02 | -3.38 | 5.6E-06 | -1.57 | HIG1 hypoxia inducible domain family member 1B |
| HIKESHI | 1.67E-02 | -3.29 | 1.2E-08 | 1.19 | heat shock protein nuclear import factor hikeshi |
| HJV | 1.29E-04 | 4.78 | 2.5E-02 | -1.28 | hemojuvelin BMP co-receptor |
| HLF | 1.49E-02 | -3.34 | 2.6E-08 | 1.02 | HLF transcription factor, PAR bZIP family member |
| HS6ST2 | 3.82E-02 | 2.95 | 1.4E-02 | 1.12 | heparan sulfate 6-O-sulfotransferase 2 |
| HSP90B1 | 3.66E-04 | 4.50 | 4.6E-09 | -1.24 | heat shock protein 90 beta family member 1 |
| HSPB8 | 1.37E-02 | 3.37 | 2.6E-07 | -1.01 | heat shock protein family B (small) member 8 |
| ID4 | 6.52E-03 | 3.63 | 5.0E-10 | -1.19 | inhibitor of DNA binding 4, HLH protein |
| IDNK | 2.11E-02 | 3.20 | 2.9E-07 | 1.07 | IDNK gluconokinase |
| IGFBPL1 | 1.56E-04 | -4.73 | 1.5E-08 | 1.22 | insulin like growth factor binding protein like 1 |
| IK | 4.26E-02 | 2.90 | 2.9E-09 | 1.01 | IK cytokine |
| IL17RB | 4.43E-02 | 2.89 | 3.4E-09 | -1.00 | interleukin 17 receptor B |
| IL31RA | 3.08E-06 | 5.68 | 2.6E-02 | 1.44 | interleukin 31 receptor A |
| JAK2 | 3.76E-02 | 2.96 | 7.6E-03 | -1.05 | Janus kinase 2 |
| JAZF1 | 6.18E-05 | 4.98 | 1.9E-14 | 1.43 | JAZF zinc finger 1 |
| JPX | 7.58E-05 | -4.93 | 1.3E-12 | -1.50 | JPX transcript, XIST activator |
| KBTBD11 | 3.09E-02 | 3.04 | 2.1E-12 | 1.08 | kelch repeat and BTB domain containing 11 |
| KCNJ16 | 2.95E-02 | 3.06 | 2.0E-08 | -1.13 | potassium inwardly rectifying channel subfamily J member 16 |
| KIN | 1.75E-02 | 3.27 | 9.4E-03 | 1.90 | Kin17 DNA and RNA binding protein |
| KIZ | 4.54E-04 | 4.45 | 9.5E-10 | -0.27 | kizuna centrosomal protein |
| KMT2E | 1.88E-04 | -4.69 | 7.3E-11 | -1.16 | lysine methyltransferase 2E (inactive) |
| KRT31 | 7.77E-04 | -4.30 | 1.3E-02 | -1.35 | keratin 31 |
| KRT33A | 1.54E-05 | -5.32 | 8.4E-03 | 1.59 | keratin 33A |
| LDHA | 2.01E-02 | -3.22 | 3.8E-14 | 1.59 | lactate dehydrogenase A |
| LHX5 | 6.95E-03 | 3.61 | 2.8E-02 | 1.34 | LIM homeobox 5 |
| LIMA1 | 5.63E-04 | 4.39 | 9.4E-09 | -1.05 | LIM domain and actin binding 1 |
| LINC00645 | 4.44E-04 | 4.45 | 3.2E-02 | 1.55 | long intergenic non-protein coding RNA 645 |
| LINC01000 | 1.05E-02 | 3.46 | 1.2E-09 | -1.43 | long intergenic non-protein coding RNA 1000 |
| LINC01133 | 6.49E-03 | 3.64 | 3.5E-02 | 1.17 | long intergenic non-protein coding RNA 1133 |
| LINC-PINT | 7.21E-05 | -4.94 | 2.6E-02 | -1.75 | long intergenic non-protein coding RNA, p53 induced transcript |
| LMOD1 | 1.71E-03 | -4.06 | 9.7E-03 | -1.91 | leiomodin 1 |
| LRFN5 | 1.82E-02 | 3.26 | 2.0E-14 | 1.11 | leucine rich repeat and fibronectin type III domain containing 5 |
| LRPPRC | 3.76E-02 | 2.96 | 1.1E-14 | 1.12 | leucine rich pentatricopeptide repeat containing |
| LRRC32 | 1.74E-02 | 3.27 | 2.5E-05 | -1.36 | leucine rich repeat containing 32 |
| LRRC39 | 8.06E-04 | -4.29 | 3.9E-02 | -1.16 | leucine rich repeat containing 39 |
| LRRC55 | 4.07E-03 | 3.79 | 3.5E-02 | -1.00 | leucine rich repeat containing 55 |
| LRRC69 | 1.67E-02 | 3.29 | 1.3E-05 | -1.07 | leucine rich repeat containing 69 |
| LUC7L3 | 2.50E-05 | 5.21 | 3.5E-12 | -1.20 | LUC7 like 3 pre-mRNA splicing factor |
| LUM | 3.25E-03 | 3.86 | 1.0E-04 | -1.22 | lumican |
| MACF1 | 4.07E-02 | -2.92 | 2.4E-08 | -1.24 | microtubule actin crosslinking factor 1 |
| MACROH2A1 | 1.83E-02 | 3.25 | 1.9E-04 | 1.10 | macroH2A.1 histone |
| MAD2L1BP | 5.38E-08 | 6.48 | 8.7E-11 | 1.12 | MAD2L1 binding protein |
| MAGED1 | 5.62E-04 | 4.39 | 1.8E-13 | 1.28 | MAGE family member D1 |
| MAGED2 | 1.09E-04 | 4.83 | 3.9E-09 | 1.50 | MAGE family member D2 |
| MALT1 | 8.11E-04 | 4.29 | 8.6E-03 | -1.46 | MALT1 paracaspase |
| MAP1B | 3.31E-02 | 3.01 | 1.2E-06 | -1.03 | microtubule associated protein 1B |
| MBD4 | 2.32E-03 | 3.97 | 4.0E-08 | 1.19 | methyl-CpG binding domain 4, DNA glycosylase |
| MBNL1 | 3.74E-02 | -2.96 | 3.6E-02 | 1.11 | muscleblind like splicing regulator 1 |
| MCCC1 | 1.69E-03 | 4.06 | 1.5E-03 | -1.01 | methylcrotonyl-CoA carboxylase subunit 1 |
| MDH1 | 2.19E-03 | 3.98 | 5.3E-14 | 1.83 | malate dehydrogenase 1 |
| MDM2 | 3.87E-03 | -3.80 | 1.8E-02 | -1.12 | MDM2 proto-oncogene |
| MDM4 | 1.24E-03 | 4.17 | 4.2E-09 | -1.33 | MDM4 regulator of p53 |
| MECR | 1.10E-03 | 4.20 | 2.4E-10 | 1.31 | mitochondrial trans-2-enoyl-CoA reductase |
| MED13 | 4.66E-02 | 2.86 | 2.2E-06 | -1.25 | mediator complex subunit 13 |
| MED6 | 9.18E-04 | 4.25 | 1.3E-05 | 1.19 | mediator complex subunit 6 |
| MFAP4 | 1.09E-04 | 4.83 | 7.2E-04 | -1.95 | microfibril associated protein 4 |
| MGST1 | 4.08E-04 | 4.48 | 1.7E-07 | -1.27 | microsomal glutathione S-transferase 1 |
| MIB1 | 1.35E-03 | -4.14 | 1.3E-07 | -1.01 | MIB E3 ubiquitin protein ligase 1 |
| MIR6840 | 1.17E-02 | 3.43 | 5.3E-03 | -1.41 | microRNA 6840 |
| MMADHC | 6.92E-04 | 4.33 | 1.3E-10 | 1.20 | metabolism of cobalamin associated D |
| MON2 | 9.93E-04 | -4.23 | 1.1E-02 | 1.40 | MON2 homolog, regulator of endosome-to-Golgi trafficking |
| MORF4L2 | 4.82E-02 | -2.85 | 3.8E-07 | -1.05 | mortality factor 4 like 2 |
| MRPL15 | 1.55E-02 | -3.32 | 3.7E-07 | 1.40 | mitochondrial ribosomal protein L15 |
| MRPL21 | 4.57E-02 | -2.87 | 2.0E-03 | -1.38 | mitochondrial ribosomal protein L21 |
| MRPL4 | 1.05E-02 | -3.46 | 1.0E-07 | 1.16 | mitochondrial ribosomal protein L4 |
| MRPS12 | 7.55E-03 | -3.59 | 6.0E-08 | 1.46 | mitochondrial ribosomal protein S12 |
| MRPS26 | 5.00E-02 | 2.83 | 3.8E-02 | 1.27 | mitochondrial ribosomal protein S26 |
| MRPS9 | 9.99E-03 | 3.48 | 1.4E-12 | 1.09 | mitochondrial ribosomal protein S9 |
| MSANTD3-TMEFF1 | 2.57E-02 | 3.12 | 3.0E-02 | 1.20 | MSANTD3-TMEFF1 readthrough |
| MT1F | 2.26E-04 | -4.64 | 2.0E-09 | -1.05 | metallothionein 1F |
| MT1X | 4.73E-02 | -2.86 | 6.3E-09 | -1.01 | metallothionein 1X |
| MTX2 | 1.68E-04 | 4.72 | 1.1E-14 | 1.47 | metaxin 2 |
| MVB12A | 1.57E-02 | 3.31 | 7.5E-03 | -1.28 | multivesicular body subunit 12A |
| MYH8 | 5.71E-03 | -3.68 | 2.1E-02 | -1.09 | myosin heavy chain 8 |
| MYL12A | 6.38E-04 | 4.35 | 9.3E-03 | -1.54 | myosin light chain 12A |
| MYLPF | 1.18E-02 | -3.42 | 5.2E-03 | -1.18 | myosin light chain, phosphorylatable, fast skeletal muscle |
| MYOF | 2.11E-05 | 5.25 | 1.7E-03 | -1.08 | myoferlin |
| NAA38 | 1.46E-04 | -4.75 | 5.1E-03 | -1.08 | N-alpha-acetyltransferase 38, NatC auxiliary subunit |
| NAP1L2 | 6.09E-03 | -3.66 | 2.0E-07 | 1.09 | nucleosome assembly protein 1 like 2 |
| NCBP2AS2 | 3.14E-02 | -3.04 | 2.6E-12 | 1.04 | NCBP2 antisense 2 (head to head) |
| NDRG4 | 1.72E-04 | -4.71 | 1.3E-13 | 1.22 | NDRG family member 4 |
| NDUFAB1 | 5.81E-03 | -3.68 | 3.6E-16 | 1.34 | NADH:ubiquinone oxidoreductase subunit AB1 |
| NDUFB10 | 3.08E-03 | -3.88 | 5.7E-09 | 1.07 | NADH:ubiquinone oxidoreductase subunit B10 |
| NDUFB5 | 2.96E-05 | -5.17 | 3.2E-12 | 1.20 | NADH:ubiquinone oxidoreductase subunit B5 |
| NDUFS3 | 7.17E-03 | 3.60 | 8.3E-10 | 1.09 | NADH:ubiquinone oxidoreductase core subunit S3 |
| NDUFV2 | 1.58E-04 | 4.73 | 4.8E-10 | 1.11 | NADH:ubiquinone oxidoreductase core subunit V2 |
| NECAP1 | 1.90E-02 | 3.24 | 1.4E-13 | 1.51 | NECAP endocytosis associated 1 |
| NFAT5 | 1.56E-02 | -3.32 | 7.1E-11 | -1.20 | nuclear factor of activated T cells 5 |
| NFYA | 3.77E-02 | 2.96 | 8.2E-03 | -0.33 | nuclear transcription factor Y subunit alpha |
| NHP2 | 1.64E-02 | -3.30 | 8.2E-14 | 1.01 | NHP2 ribonucleoprotein |
| NIT1 | 5.03E-04 | 4.42 | 5.0E-03 | -1.51 | nitrilase 1 |
| NKIRAS2 | 1.58E-03 | -4.09 | 1.3E-02 | -1.47 | NFKB inhibitor interacting Ras like 2 |
| NMT2 | 1.69E-03 | 4.07 | 7.0E-08 | -1.03 | N-myristoyltransferase 2 |
| NOP16 | 4.71E-03 | 3.74 | 3.5E-02 | 1.28 | NOP16 nucleolar protein |
| NPIPA5 | 3.44E-05 | -5.13 | 1.1E-07 | -1.24 | nuclear pore complex interacting protein family member A5 |
| NPNT | 1.00E-02 | -3.48 | 3.5E-05 | -1.02 | nephronectin |
| NT5M | 1.15E-02 | 3.43 | 6.0E-03 | 1.26 | 5',3'-nucleotidase, mitochondrial |
| NUCB1 | 4.83E-03 | 3.73 | 4.8E-02 | -1.16 | nucleobindin 1 |
| NUDT21 | 7.49E-04 | 4.31 | 1.1E-02 | 1.13 | nudix hydrolase 21 |
| NUP58 | 3.63E-04 | 4.51 | 2.1E-04 | -1.61 | nucleoporin 58 |
| NUP98 | 4.08E-04 | -4.47 | 1.1E-02 | -1.57 | nucleoporin 98 and 96 precursor |
| OARD1 | 2.53E-03 | -3.94 | 3.3E-02 | 1.07 | O-acyl-ADP-ribose deacylase 1 |
| OPN3 | 5.05E-10 | 7.27 | 1.4E-06 | 1.09 | opsin 3 |
| OR7E47P | 2.92E-02 | -3.07 | 7.0E-06 | 1.02 | olfactory receptor family 7 subfamily E member 47 pseudogene |
| OXCT1 | 5.71E-03 | 3.68 | 2.6E-09 | 1.19 | 3-oxoacid CoA-transferase 1 |
| OXLD1 | 5.03E-04 | 4.42 | 8.4E-09 | 1.24 | oxidoreductase like domain containing 1 |
| P2RY14 | 3.99E-03 | 3.79 | 1.0E-05 | -1.16 | purinergic receptor P2Y14 |
| PAAF1 | 3.91E-06 | -5.63 | 9.7E-09 | -1.05 | proteasomal ATPase associated factor 1 |
| PAFAH1B1 | 8.55E-06 | 5.46 | 3.6E-06 | 1.20 | platelet activating factor acetylhydrolase 1b regulatory subunit 1 |
| PAQR4 | 4.61E-05 | 5.06 | 6.7E-03 | -1.51 | progestin and adipoQ receptor family member 4 |
| PARM1 | 1.31E-03 | 4.15 | 5.3E-07 | 1.50 | prostate androgen-regulated mucin-like protein 1 |
| PCDHGA4 | 4.73E-02 | -2.86 | 3.7E-13 | -1.06 | protocadherin gamma subfamily A, 4 |
| PCSK5 | 3.16E-02 | -3.03 | 2.2E-10 | -1.26 | proprotein convertase subtilisin/kexin type 5 |
| PDHB | 3.53E-02 | -2.99 | 8.7E-10 | 1.29 | pyruvate dehydrogenase E1 subunit beta |
| PEAR1 | 4.12E-02 | 2.92 | 1.3E-06 | -1.15 | platelet endothelial aggregation receptor 1 |
| PFKM | 2.81E-03 | 3.91 | 1.9E-14 | 1.24 | phosphofructokinase, muscle |
| PGK1 | 5.78E-03 | -3.68 | 4.7E-07 | 1.28 | phosphoglycerate kinase 1 |
| PGM3 | 2.62E-03 | 3.93 | 1.3E-04 | 1.01 | phosphoglucomutase 3 |
| PHYHD1 | 2.44E-03 | 3.95 | 6.8E-08 | -1.11 | phytanoyl-CoA dioxygenase domain containing 1 |
| PLA1A | 2.23E-09 | 7.04 | 6.2E-04 | -1.04 | phospholipase A1 member A |
| PLA2G2A | 3.74E-03 | 3.82 | 1.2E-02 | -1.14 | phospholipase A2 group IIA |
| PLGLB1 | 2.85E-02 | -3.08 | 6.7E-13 | -1.44 | plasminogen like B1 |
| PLSCR4 | 3.80E-02 | 2.95 | 1.0E-12 | -1.28 | phospholipid scramblase 4 |
| PLXNB1 | 5.00E-02 | -2.83 | 6.2E-08 | -1.52 | plexin B1 |
| PNMA8A | 2.73E-03 | 3.92 | 9.2E-10 | 1.31 | PNMA family member 8A |
| PNMA8B | 1.11E-02 | 3.44 | 4.4E-09 | 1.42 | PNMA family member 8B |
| POGLUT3 | 6.05E-03 | 3.66 | 6.7E-07 | -1.03 | protein O-glucosyltransferase 3 |
| POLB | 1.16E-02 | 3.43 | 3.3E-02 | -1.71 | DNA polymerase beta |
| POM121C | 2.42E-03 | 3.96 | 3.1E-02 | -1.34 | POM121 transmembrane nucleoporin C |
| POT1 | 2.09E-02 | 3.20 | 2.2E-02 | 1.05 | protection of telomeres 1 |
| PPL | 8.61E-06 | 5.45 | 4.0E-02 | 1.26 | periplakin |
| PPP2CA | 4.01E-02 | 2.93 | 2.0E-11 | 1.23 | protein phosphatase 2 catalytic subunit alpha |
| PPP2R3C | 2.39E-02 | -3.15 | 5.4E-08 | -1.28 | protein phosphatase 2 regulatory subunit B''gamma |
| PREPL | 1.16E-04 | 4.81 | 3.0E-08 | 1.20 | prolyl endopeptidase like |
| PRKAG1 | 8.63E-03 | 3.54 | 1.8E-07 | 1.08 | protein kinase AMP-activated non-catalytic subunit gamma 1 |
| PRKRA | 7.93E-03 | 3.56 | 1.4E-02 | -1.09 | protein activator of interferon induced protein kinase EIF2AK2 |
| PRNP | 5.76E-05 | 5.00 | 1.8E-02 | 1.23 | prion protein |
| PRPF38B | 2.01E-02 | -3.22 | 3.0E-05 | -1.11 | pre-mRNA processing factor 38B |
| PSMA5 | 1.84E-02 | 3.25 | 1.8E-12 | 1.34 | proteasome 20S subunit alpha 5 |
| PSMA7 | 1.44E-02 | 3.35 | 2.0E-06 | 1.03 | proteasome 20S subunit alpha 7 |
| PSMA8 | 3.77E-03 | 3.81 | 4.3E-02 | -1.59 | proteasome 20S subunit alpha 8 |
| PSMB1 | 2.07E-02 | -3.21 | 3.3E-05 | 1.06 | proteasome 20S subunit beta 1 |
| PSMC2 | 2.47E-04 | -4.62 | 5.4E-09 | 1.04 | proteasome 26S subunit, ATPase 2 |
| PSMD12 | 1.54E-03 | 4.10 | 1.8E-12 | 1.15 | proteasome 26S subunit, non-ATPase 12 |
| PSMD8 | 4.67E-03 | 3.74 | 4.6E-10 | 1.68 | proteasome 26S subunit, non-ATPase 8 |
| PTGFR | 7.17E-03 | -3.60 | 3.9E-02 | 1.24 | prostaglandin F receptor |
| PTPRK | 6.48E-03 | 3.64 | 2.0E-03 | 1.55 | protein tyrosine phosphatase receptor type K |
| PTRH2 | 4.02E-06 | 5.62 | 7.9E-09 | 1.20 | peptidyl-tRNA hydrolase 2 |
| QKI | 3.59E-02 | -2.98 | 2.0E-08 | -1.24 | QKI, KH domain containing RNA binding |
| RAB11A | 1.58E-02 | 3.31 | 2.0E-12 | 1.18 | RAB11A, member RAS oncogene family |
| RAB11B-AS1 | 3.49E-04 | 4.52 | 4.0E-02 | -1.24 | RAB11B antisense RNA 1 |
| RAB11FIP3 | 5.61E-05 | 5.01 | 4.7E-14 | -1.58 | RAB11 family interacting protein 3 |
| RAB38 | 2.13E-02 | 3.19 | 5.9E-03 | -1.39 | RAB38, member RAS oncogene family |
| RAD51AP1 | 2.93E-03 | 3.89 | 9.8E-03 | -1.76 | RAD51 associated protein 1 |
| RALYL | 7.20E-05 | 4.94 | 5.0E-06 | 1.16 | RALY RNA binding protein like |
| RASA1 | 1.01E-02 | 3.48 | 7.2E-11 | 1.17 | RAS p21 protein activator 1 |
| RBBP4 | 1.76E-02 | 3.27 | 1.4E-09 | -1.08 | RB binding protein 4, chromatin remodeling factor |
| RBM25 | 3.71E-02 | 2.96 | 6.5E-12 | -1.52 | RNA binding motif protein 25 |
| RBM5 | 4.12E-02 | 2.92 | 1.2E-07 | -1.18 | RNA binding motif protein 5 |
| RERG | 1.57E-06 | -5.85 | 2.8E-02 | 1.52 | RAS like estrogen regulated growth inhibitor |
| REST | 2.50E-03 | 3.95 | 6.1E-11 | -1.24 | RE1 silencing transcription factor |
| RIIAD1 | 3.97E-02 | 2.93 | 3.3E-09 | 1.08 | regulatory subunit of type II PKA R-subunit domain containing 1 |
| RLIM | 7.49E-04 | 4.31 | 8.1E-03 | -1.19 | ring finger protein, LIM domain interacting |
| RNASE6 | 2.76E-03 | 3.91 | 1.1E-02 | -1.29 | ribonuclease A family member k6 |
| RNF141 | 8.02E-03 | 3.56 | 1.3E-05 | 1.01 | ring finger protein 141 |
| RNPC3 | 2.35E-02 | 3.16 | 4.4E-13 | -1.24 | RNA binding region (RNP1, RRM) containing 3 |
| ROBO2 | 2.79E-02 | 3.09 | 1.3E-07 | 1.12 | roundabout guidance receptor 2 |
| RORB | 3.63E-04 | 4.51 | 1.9E-03 | 1.02 | RAR related orphan receptor B |
| RP2 | 9.85E-03 | 3.49 | 6.0E-03 | 1.96 | RP2 activator of ARL3 GTPase |
| RRAGB | 9.94E-03 | 3.48 | 3.4E-11 | 1.67 | Ras related GTP binding B |
| RSBN1 | 7.77E-03 | -3.57 | 1.1E-03 | 2.46 | round spermatid basic protein 1 |
| RTN3 | 1.92E-02 | 3.24 | 7.3E-16 | 1.77 | reticulon 3 |
| RTRAF | 2.54E-02 | -3.13 | 1.0E-09 | 1.05 | RNA transcription, translation and transport factor |
| SAP18 | 2.83E-03 | 3.90 | 1.8E-15 | 1.64 | Sin3A associated protein 18 |
| SARS1 | 1.38E-03 | 4.13 | 3.1E-18 | 1.36 | seryl-tRNA synthetase 1 |
| SAT1 | 3.54E-02 | 2.99 | 7.0E-08 | -1.10 | spermidine/spermine N1-acetyltransferase 1 |
| SCG2 | 2.48E-02 | 3.14 | 7.5E-09 | 1.17 | secretogranin II |
| SCG5 | 3.78E-03 | 3.81 | 2.7E-09 | 1.29 | secretogranin V |
| SCN2A | 6.13E-06 | 5.52 | 9.3E-08 | 1.04 | sodium voltage-gated channel alpha subunit 2 |
| SDC4 | 9.88E-03 | 3.49 | 3.6E-08 | -1.02 | syndecan 4 |
| SDR16C5 | 2.72E-02 | 3.10 | 1.6E-14 | 1.67 | short chain dehydrogenase/reductase family 16C member 5 |
| SEC31B | 1.44E-02 | 3.35 | 1.9E-02 | 1.03 | SEC31 homolog B, COPII coat complex component |
| SELENOP | 3.70E-02 | -2.97 | 3.0E-05 | 0.23 | selenoprotein P |
| SEPTIN10 | 3.21E-02 | -3.03 | 1.1E-02 | -1.57 | septin 10 |
| SERPINF1 | 8.74E-03 | 3.53 | 6.9E-10 | 1.38 | serpin family F member 1 |
| SF3B5 | 2.24E-02 | 3.17 | 3.1E-16 | 1.17 | splicing factor 3b subunit 5 |
| SHPRH | 6.30E-03 | 3.65 | 4.4E-02 | 1.11 | SNF2 histone linker PHD RING helicase |
| SIGLEC16 | 1.79E-02 | 3.26 | 5.4E-03 | -1.75 | sialic acid binding Ig like lectin 16 |
| SIPA1L1 | 1.81E-03 | 4.04 | 2.3E-12 | 1.17 | signal induced proliferation associated 1 like 1 |
| SIVA1 | 3.52E-02 | 2.99 | 1.3E-06 | 0.05 | SIVA1 apoptosis inducing factor |
| SLC15A2 | 8.72E-05 | -4.89 | 7.1E-10 | -1.24 | solute carrier family 15 member 2 |
| SLC16A6 | 1.68E-02 | 3.29 | 1.2E-07 | 1.00 | solute carrier family 16 member 6 |
| SLC25A12 | 6.82E-04 | 4.34 | 2.7E-12 | 1.31 | solute carrier family 25 member 12 |
| SLC25A13 | 1.17E-03 | 4.18 | 8.0E-08 | -1.13 | solute carrier family 25 member 13 |
| SLC25A3 | 8.65E-04 | 4.27 | 1.5E-09 | 1.11 | solute carrier family 25 member 3 |
| SLC25A4 | 5.22E-03 | 3.71 | 2.6E-14 | 1.31 | solute carrier family 25 member 4 |
| SLC44A5 | 1.78E-02 | -3.27 | 1.2E-02 | 1.84 | solute carrier family 44 member 5 |
| SLF1 | 2.15E-05 | 5.24 | 2.3E-04 | 1.53 | SMC5-SMC6 complex localization factor 1 |
| SLIRP | 4.26E-03 | 3.77 | 8.9E-16 | 1.60 | SRA stem-loop interacting RNA binding protein |
| SLIT2 | 5.04E-03 | 3.72 | 3.2E-11 | 1.44 | slit guidance ligand 2 |
| SLITRK4 | 7.09E-03 | 3.61 | 1.2E-04 | 1.13 | SLIT and NTRK like family member 4 |
| SMAD9 | 1.71E-04 | 4.71 | 3.1E-02 | -1.15 | SMAD family member 9 |
| SMC4 | 1.55E-03 | 4.09 | 4.3E-02 | -1.11 | structural maintenance of chromosomes 4 |
| SNRPA1 | 4.80E-02 | 2.85 | 3.5E-10 | 1.61 | small nuclear ribonucleoprotein polypeptide A' |
| SNX10 | 1.27E-03 | 4.16 | 4.1E-11 | 1.41 | sorting nexin 10 |
| SNX14 | 3.09E-02 | 3.04 | 2.1E-11 | 1.23 | sorting nexin 14 |
| SOX9 | 2.01E-03 | 4.01 | 7.2E-10 | -1.06 | SRY-box transcription factor 9 |
| SPPL2A | 7.15E-04 | 4.32 | 2.2E-06 | 0.16 | signal peptide peptidase like 2A |
| SRRM2 | 7.51E-04 | 4.31 | 8.6E-13 | -1.49 | serine/arginine repetitive matrix 2 |
| SSBP3-AS1 | 1.45E-03 | 4.11 | 2.3E-10 | -1.17 | SSBP3 antisense RNA 1 |
| STAG1 | 4.30E-02 | 2.90 | 2.0E-05 | -1.03 | stromal antigen 1 |
| STAG2 | 1.12E-03 | 4.19 | 1.4E-11 | -1.41 | stromal antigen 2 |
| STEAP2 | 3.18E-02 | 3.03 | 3.5E-09 | 1.09 | STEAP2 metalloreductase |
| STRIP2 | 1.40E-03 | 4.13 | 3.2E-02 | -1.52 | striatin interacting protein 2 |
| SUSD1 | 4.06E-07 | 6.12 | 7.0E-06 | 1.13 | sushi domain containing 1 |
| TAF15 | 3.43E-04 | 4.52 | 1.2E-10 | -1.34 | TATA-box binding protein associated factor 15 |
| TCEAL7 | 1.72E-02 | -3.28 | 3.8E-08 | 1.16 | transcription elongation factor A like 7 |
| TGFBR3 | 2.70E-02 | 3.10 | 4.1E-07 | -1.16 | transforming growth factor beta receptor 3 |
| THEM6 | 3.08E-03 | 3.88 | 2.5E-07 | 1.24 | thioesterase superfamily member 6 |
| TIPIN | 3.66E-02 | 2.97 | 3.2E-02 | -1.30 | TIMELESS interacting protein |
| TJP2 | 7.53E-03 | -3.59 | 2.8E-05 | -1.13 | tight junction protein 2 |
| TLE4 | 1.17E-04 | 4.81 | 8.1E-10 | -1.37 | TLE family member 4, transcriptional corepressor |
| TMEM141 | 1.44E-02 | 3.35 | 1.6E-02 | 1.22 | transmembrane protein 141 |
| TMEM158 | 2.06E-04 | 4.66 | 2.8E-02 | 1.11 | transmembrane protein 158 |
| TMEM200A | 2.49E-02 | 3.13 | 3.7E-06 | 1.19 | transmembrane protein 200A |
| TMEM260 | 3.76E-02 | -2.96 | 1.4E-02 | -1.36 | transmembrane protein 260 |
| TMEM35A | 1.89E-03 | -4.03 | 9.4E-11 | 1.02 | transmembrane protein 35A |
| TMEM59L | 1.15E-02 | -3.43 | 1.9E-07 | 1.15 | transmembrane protein 59 like |
| TMTC4 | 1.82E-06 | 5.81 | 9.5E-03 | -1.33 | transmembrane O-mannosyltransferase targeting cadherins 4 |
| TOMM5 | 4.73E-02 | 2.86 | 6.7E-11 | 1.05 | translocase of outer mitochondrial membrane 5 |
| TOP1 | 3.22E-02 | -3.03 | 5.7E-08 | -1.04 | DNA topoisomerase I |
| TOR1AIP2 | 8.11E-04 | 4.29 | 3.8E-02 | 1.01 | torsin 1A interacting protein 2 |
| TP53TG1 | 4.53E-03 | 3.75 | 7.8E-03 | -1.35 | TP53 target 1 |
| TRIM33 | 3.31E-02 | -3.01 | 5.8E-07 | -1.15 | tripartite motif containing 33 |
| TRIM38 | 4.27E-03 | 3.77 | 9.2E-05 | -1.26 | tripartite motif containing 38 |
| TRMT10C | 2.92E-02 | 3.07 | 6.8E-10 | 1.16 | tRNA methyltransferase 10C, mitochondrial RNase P subunit |
| TRPM7 | 2.77E-02 | -3.09 | 7.6E-03 | -1.09 | transient receptor potential cation channel subfamily M member 7 |
| TRPS1 | 4.31E-03 | 3.77 | 3.1E-05 | -1.07 | transcriptional repressor GATA binding 1 |
| TUBB4B | 3.22E-02 | 3.02 | 1.9E-20 | 1.69 | tubulin beta 4B class IVb |
| UGP2 | 5.55E-05 | -5.01 | 2.0E-09 | 1.11 | UDP-glucose pyrophosphorylase 2 |
| UHRF1 | 4.65E-02 | -2.86 | 6.3E-07 | -1.27 | ubiquitin like with PHD and ring finger domains 1 |
| UQCR10 | 1.31E-06 | -5.88 | 5.3E-13 | 1.09 | ubiquinol-cytochrome c reductase, complex III subunit X |
| UQCRC1 | 1.55E-02 | 3.32 | 1.1E-11 | 1.64 | ubiquinol-cytochrome c reductase core protein 1 |
| UQCRFS1 | 5.60E-03 | 3.69 | 9.9E-15 | 1.36 | ubiquinol-cytochrome c reductase, Rieske iron-sulfur polypeptide 1 |
| USP32 | 1.10E-02 | 3.45 | 2.0E-02 | -1.18 | ubiquitin specific peptidase 32 |
| USP34 | 1.34E-02 | 3.38 | 3.6E-14 | -1.23 | ubiquitin specific peptidase 34 |
| USP40 | 9.00E-03 | 3.52 | 1.6E-02 | -1.18 | ubiquitin specific peptidase 40 |
| VCAN | 1.47E-02 | 3.34 | 1.9E-12 | -1.24 | versican |
| VEGFC | 4.05E-02 | 2.92 | 4.9E-02 | 1.31 | vascular endothelial growth factor C |
| VSNL1 | 9.44E-03 | -3.50 | 1.4E-10 | 1.84 | visinin like 1 |
| WASF2 | 2.86E-02 | 3.08 | 6.5E-06 | -1.25 | WASP family member 2 |
| WASHC3 | 6.38E-04 | 4.35 | 2.6E-11 | 1.10 | WASH complex subunit 3 |
| WDR61 | 4.63E-02 | 2.87 | 6.4E-11 | 1.75 | WD repeat domain 61 |
| WNT5A | 2.16E-03 | -3.99 | 3.3E-02 | 1.07 | Wnt family member 5A |
| YAP1 | 6.06E-03 | 3.66 | 2.0E-09 | -1.25 | Yes1 associated transcriptional regulator |
| YKT6 | 8.97E-04 | 4.26 | 3.4E-09 | 1.60 | YKT6 v-SNARE homolog |
| YWHAZ | 2.64E-02 | 3.11 | 3.5E-15 | 1.52 | tyrosine 3-monooxygenase/tryptophan 5-monooxygenase activation protein zeta |
| ZBTB20 | 1.64E-02 | 3.30 | 1.6E-08 | -1.19 | zinc finger and BTB domain containing 20 |
| ZC3H14 | 7.54E-03 | 3.59 | 5.7E-09 | 1.10 | zinc finger CCCH-type containing 14 |
| ZFAND6 | 4.47E-03 | -3.76 | 4.6E-08 | -1.31 | zinc finger AN1-type containing 6 |
| ZHX2 | 1.25E-02 | 3.40 | 8.2E-04 | -1.08 | zinc fingers and homeoboxes 2 |
| ZNF174 | 4.29E-02 | 2.90 | 1.3E-02 | 1.29 | zinc finger protein 174 |
| ZNF217 | 3.26E-05 | 5.14 | 6.4E-07 | -1.06 | zinc finger protein 217 |
| ZNF222 | 1.14E-03 | 4.19 | 7.4E-08 | 1.31 | zinc finger protein 222 |
| ZNF367 | 3.02E-04 | 4.56 | 3.1E-02 | -1.36 | zinc finger protein 367 |
| ZNF385B | 1.40E-03 | 4.13 | 4.2E-04 | 0.23 | zinc finger protein 385B |
| ZNF566 | 9.15E-04 | 4.25 | 1.1E-09 | -1.30 | zinc finger protein 566 |
| ZNF569 | 1.03E-04 | 4.84 | 9.0E-03 | -1.70 | zinc finger protein 569 |
| ZNF711 | 2.67E-02 | 3.11 | 1.7E-03 | 1.17 | zinc finger protein 711 |
| ZNF721 | 5.98E-03 | 3.66 | 1.4E-11 | -1.15 | zinc finger protein 721 |
| ZNF770 |  |  | 1.1E-02 | -1.19 | zinc finger protein 770 |
| ZSCAN30 |  |  | 8.4E-06 | -1.22 | zinc finger and SCAN domain containing 30 |

**Table S5.** Five highest-ranked clustering gene modules in the protein-protein interaction network of differentially expressed genes of musculoskeletal aging and Alzheimer’s disease.

| **Cluster** | **MCODE Score** | **Gene density** | **Gene edges** |
| --- | --- | --- | --- |
| *Musculoskeletal Aging* | | | |
| 1 | 32.542 | 60 | 960 |
| 2 | 17.000 | 19 | 153 |
| 3 | 8.278 | 116 | 476 |
| 4 | 8.019 | 109 | 433 |
| 5 | 6.286 | 36 | 110 |
|  |  |  |  |
| *Alzheimer’s disease* | | | |
| 1 | 24.043 | 186 | 2224 |
| 2 | 20.025 | 81 | 801 |
| 3 | 14.774 | 244 | 1795 |
| 4 | 7.417 | 128 | 471 |
| 5 | 6.286 | 8 | 22 |

**Table S6.** Gene composition of the highest-ranked clustering modules in the protein-protein interaction network of differentially expressed genes of musculoskeletal aging and Alzheimer’s disease.

| **Cluster** | **Genes** |
| --- | --- |
| *Musculoskeletal aging* | |
| 1 | ATP5F1D, ATP5MC3, ATP5PF, AURKAIP1, CHCHD1, COA6, COX4I1, COX6B1, COX6C, COX7B, COX7C, CYC1, MRPL11, MRPL12, MRPL15, MRPL18, MRPL19, MRPL21, MRPL3, MRPL33, MRPL34, MRPL35, MRPL36, MRPL39, MRPL4, MRPL41, MRPL46, MRPL48, MRPL51, MRPS12, MRPS16, MRPS17, MRPS22, MRPS24, MRPS26, MRPS33, MRPS9, NDUFA12, NDUFA3, NDUFA6, NDUFA9, NDUFAB1, NDUFB10, NDUFB11, NDUFB5, NDUFB6, NDUFB9, NDUFC1, NDUFS3, NDUFS4, NDUFS6, NDUFV2, PTCD3, SDHC, UQCR10, UQCR11, UQCRC1, UQCRFS1, UQCRH, UQCRQ |
| 2 | C18orf32, EEF1G, EIF1AX, EPRS1, MTRF1, NHP2, RPL17, RPL23A, RPL26L1, RPL30, RPL36A, RPL3L, RPLP0, RPS10, RPS18, RPS3, RPSA, RSL24D1, UBXN7 |
|  | |
| *Alzheimer’s disease* | |
| 1 | ACLY, ACTA2, ACTB, ALB, ANGPT2, APOBEC3G, AR, AREG, ARG1, ATP5F1A, ATP5F1C, ATP5MF-PTCD1, ATP6AP1, ATP6V0A1, ATP6V0A2, ATR, ATRX, BARD1, BMP2, BMP7, BRIP1, C17orf80, CAV1, CCN2, CD163, CD19, CD24, CDC42, CDH1, COL1A1, COX7B, CTSC, CTSZ, CXCL12, CXCL2, CXCR4, CYC1, DGUOK, DMAC2L, DTL, EGFR, EIF3B, EIF3D, EIF3G, ENG, ENO1, ENO2, ERCC1, F5, FANCA, FANCD2, FANCM, FGF13, FGFR1, FH, FLT1, FN1, FOXO1, GABRG2, GADD45GIP1, GAPDH, GFM1, GJA1, GORASP1, GOSR2, GOT2, GPI, GRIA4, H6PD, HJURP, HNF4A, HUS1, IDH1, IDH3A, IDH3B, IDH3G, IGF1, IMPDH2, IRS1, ITGA6, ITGB1, JAG1, JAK2, KARS1, KITLG, LDHA, MAP2K1, MAPK1, MCM4, MDH1, MDH2, MET, MRPL15, MRPL17, MRPL21, MRPL30, MRPL37, MRPL4, MRPL43, MRPL47, MRPL9, MRPS12, MRPS15, MRPS23, MRPS26, MRPS28, MRPS30, MRPS5, MRPS9, MSH2, MSH3, MT-ND6, MTOR, NRP1, NRXN1, NTRK1, OGDH, PAAF1, PECAM1, PGD, PGF, PGK1, PGK2, PKM, PLK4, POLH, POSTN, PPA2, PPARG, PRDX3, PSMA1, PSMA3, PSMA5, PSMA7, PSMB2, PSMB7, PSMC2, PSMC3, PSMC4, PSMC5, PSMD1, PSMD11, PSMD12, PSMD14, PSMD4, RAB1A, RAD18, RAD51C, RAD51D, RAD52, RPA1, RPA3, RPL35A, RUVBL1, SCFD1, SDHB, SEC22B, SEC22C, SEC23A, SEC24A, SHC1, SLC25A3, SMAD2, SOCS3, SOX9, SST, SUCLA2, SUCLG2, SYN2, TALDO1, THBS1, TMED10, TMED2, TNPO1, TRAPPC1, TRAPPC2L, UBE2V2, UQCRC1, UQCRC2, UQCRFS1, VEGFC, VWF, WNT5A, XRCC2, XRCC6, YKT6, |
| 2 | ATP5F1B, ATP5MC3, ATP5PO, ATP6AP1L, ATP6AP2, ATP6V0B, ATP6V0C, ATP6V0E1, ATP6V1A, ATP6V1B2, ATP6V1C1, ATP6V1C2, ATP6V1D, ATP6V1E1, ATP6V1G2, ATP6V1H, CCT2, CCT4, CCT5, CCT7, CFTR, COA6, COPS5, COX5B, COX6A1, COX6B2, DLD, EIF2S3, EIF3M, FLCN, FNIP1, GLI2, GLI3, HNRNPD, IL1B, ITFG2, LAMTOR2, MPND, NDUFA1, NDUFA11, NDUFA13, NDUFA4, NDUFA7, NDUFAB1, NDUFB10, NDUFB5, NDUFB8, NDUFC2-KCTD14, NDUFS3, NDUFS7, NDUFV1, NDUFV2, NFKBIA, NPRL3, OAZ1, PRNP, PSMA8, PSMB1, PSMB3, PSMB4, PSMD10, PSMD8, PSME3, RAN, RHEB, RPL15, RPS16, RRAGA, RRAGB, RUNX3, SEH1L, SESN2, SNCA, SOD1, SPATA5, TCIRG1, UBE3A, UBLCP1, UCHL5, UQCR10, WDR59, |

**Table S7.** Five highest-ranked hub genes according to 11 topological algorithms ranked in the protein-protein interaction network of differentially expressed genes between musculoskeletal aging and Alzheimer’s disease. Numbers represent score

| **Topological Score** | **NDUFAB1** | **UQCRC1** | **UQCRFS1** | **MRPL15** | **NDUFS3** |
| --- | --- | --- | --- | --- | --- |
| *Musculoskeletal aging* | | | | | |
| MCC | 9.22E+13 | 9.22E+13 | 9.22E+13 | 9.22E+13 | 9.22E+13 |
| DMNC | 0.50 | 0.82 | 0.63 | 0.79 | 0.68 |
| MNC | 91.00 | 56.00 | 70.00 | 62.00 | 65.00 |
| Degree | 92.00 | 61.00 | 70.00 | 63.00 | 66.00 |
| EPC | 71.16 | 55.99 | 63.69 | 64.05 | 61.72 |
| BottleNeck | 9.00 | 3.00 | 1.00 | 1.00 | 1.00 |
| EcCentricity | 0.17 | 0.14 | 0.17 | 0.17 | 0.14 |
| Closeness | 694.43 | 672.93 | 689.28 | 673.68 | 679.78 |
| Radiality | 8.17 | 8.13 | 8.18 | 8.12 | 8.15 |
| Betweenness | 31333.89 | 14933.76 | 11964.04 | 5380.12 | 10473.19 |
| Stress | 443608.00 | 226458.00 | 260576.00 | 165494.00 | 221734.00 |
|  |  |  |  |  |  |
| *Alzheimer’s disease* | | | | | |
| MCC | 9.22E+13 | 9.22E+13 | 9.22E+13 | 9.22E+13 | 9.22E+13 |
| DMNC | 0.73 | 0.44 | 0.62 | 0.65 | 0.49 |
| MNC | 79.00 | 99.00 | 96.00 | 88.00 | 64.00 |
| Degree | 83.00 | 105.00 | 100.00 | 88.00 | 66.00 |
| EPC | 177.79 | 169.98 | 166.88 | 184.06 | 125.36 |
| BottleNeck | 4.00 | 5.00 | 2.00 | 1.00 | 5.00 |
| EcCentricity | 0.20 | 0.20 | 0.20 | 0.20 | 0.20 |
| Closeness | 1366.35 | 1401.70 | 1376.25 | 1383.32 | 1335.03 |
| Radiality | 6.26 | 6.32 | 6.27 | 6.29 | 6.21 |
| Betweenness | 22790.18 | 44654.13 | 22842.87 | 10235.16 | 21583.50 |
| Stress | 569708.00 | 992102.00 | 547242.00 | 394946.00 | 447902.00 |
